# ENaC phosphorylation facilitates ankyrin-3 interaction critical for renal sodium balance

**DOI:** 10.1093/pnasnexus/pgag253

**Published:** 2026-07-29

**Authors:** Tarek Mohamed Abd El-Aziz, Antonio G Soares, Elena Mironova, Crystal R Archer, Amr Setouhi, Brij B Singh, James D Stockand

**Affiliations:** Center for Regenerative Sciences, University of Texas Health Science Center at San Antonio, San Antonio, TX 78229, USA; Zoology Department, Faculty of Science, Minia University, El-Minia 61519, Egypt; Pacific Biosciences Research Center, University of Hawaiʻi at Mānoa, Honolulu, HI 96848, USA; Greehey Children's Cancer Research Institute, University of Texas Health Science Center at San Antonio, San Antonio, TX 78229, USA; Department of Chemistry and Biochemistry, University of Arkansas, Fayetteville, AR 72701, USA; Cardiology Department, Faculty of Medicine, Minia University, El-Minia 61519, Egypt; Center for Regenerative Sciences, University of Texas Health Science Center at San Antonio, San Antonio, TX 78229, USA; Department of Cellular and Integrative Physiology, University of Texas Health Science Center at San Antonio, San Antonio, TX 78229, USA

**Keywords:** epithelial sodium channel, casein kinase, phosphorylation, sodium reabsorption, salt-sensitive hypertension

## Abstract

Kidneys play a pivotal role in long-term blood pressure regulation by controlling extracellular fluid volume through renal salt reabsorption. Discretionary control of the Epithelial Na^+^ Channel, ENaC, is the final step in fine-tuning renal Na^+^ excretion. Consequently, gain-of-function mutations in ENaC result in hypertension caused by inappropriate retention of Na^+^, whereas loss-of-function of this channel causes abnormal renal salt wasting. Thus, understanding how ENaC activity is regulated is important for a complete understanding of the molecular origins of hypertension. Our data show that casein kinase 2 (CK2) phosphorylates β-ENaC at a conserved motif homologous to the CK2 site in Nav1.2 and KCNQ channels, where phosphorylation controls ankyrin-3 (Ank-3) binding and channel membrane localization. In addition, CK2-dependent phosphorylation of β-ENaC is required for Ank-3 binding, which stabilizes ENaC at the apical membrane, sustains channel activity, and thereby ensures appropriate renal Na^+^ excretion. Using structure-guided mutagenesis of the β-ENaC C-terminus combined with advanced live-cell imaging (TIRF-FRAP, FRET) and patch-clamp electrophysiology, we found that mutation of the CK2 consensus site or disruption of the Ank-3-binding motif abolishes ENaC surface mobility and suppresses macroscopic current density. Principal cell-specific deletion of CK2 or Ank-3, or pharmacologic CK2 inhibition, eliminates ENaC activity in native collecting ducts and drives markedly accelerated urinary sodium loss in vivo. These results identify CK2-dependent Ank-3 scaffolding as an essential post-translational mechanism that maintains ENaC at the apical membrane and sustains renal Na^+^ reabsorption, thereby presenting a potential new targetable regulatory pathway with implications for sodium homeostasis and blood pressure control.

Significance statement The epithelial sodium channel (ENaC) is the final arbiter of renal sodium reabsorption and a critical regulator of fluid volume and blood pressure. While hormonal pathways controlling ENaC are well characterized, the post-translational mechanisms that stabilize active channels at the apical membrane remain incompletely identified. We define a conserved phosphorylation-dependent anchor motif within the β-ENaC C-terminus that couples casein kinase 2 activity to ankyrin-3 binding. CK2-mediated phosphorylation promotes ankyrin-3 binding, enhances membrane retention, and sustains channel open probability and surface expression. Genetic deletion of this pathway abolishes ENaC activity in principal cells and induces marked natriuresis in vivo, establishing CK2/ankyrin-3 coupling as an essential regulator of renal sodium homeostasis with potential implications for understanding the sodium-sensitive blood pressure regulation.

## Introduction

Hypertension is the leading preventable risk factor for cardiovascular disease and premature death worldwide (1), and when uncontrolled, it remains a silent but devastating contributor to heart disease, stroke, and chronic kidney disease, the leading causes of mortality globally (2–4). An estimated 1.4 billion adults aged 30–79 years are currently living with hypertension worldwide, yet fewer than one in five have the condition adequately controlled (5–7). The pathophysiology of hypertension is multifactorial, involving abnormalities in vascular tone regulation, impaired endothelial function, dysregulation of neurohormonal systems, including the renin–angiotensin–aldosterone system and sympathetic nervous system, and, critically, altered renal sodium handling (8, 9). High sodium consumption is consistently associated with increased blood pressure and is recognized as a significant dietary contributor to hypertension (10–13). In the aldosterone-sensitive distal nephron, the epithelial sodium channel (ENaC) is a key regulator of epithelial Na^+^ absorption and therefore plays a pivotal role in extracellular fluid volume homeostasis and blood pressure control (14–16). ENaC belongs to the Deg/ENaC superfamily of ion channels and is composed of three homologous subunits (α, β, and γ) that assemble as heterotrimers (17–19). The clinical importance of ENaC in blood pressure regulation is exemplified by two inherited disorders. Gain-of-function mutations in ENaC cause Liddle syndrome, a severe form of hereditary hypertension characterized by inappropriate Na^+^ retention (20, 21), whereas loss-of-function mutations produce pseudohypoaldosteronism type 1, a salt-wasting disease associated with hypotension (22, 23).

ENaC activity at the plasma membrane is dynamically regulated through a complex interplay of post-translational modifications, scaffolding protein interactions, and membrane trafficking events (24). Ubiquitination by the E3 ubiquitin ligase Nedd4-2 reduces ENaC surface expression. Nedd4-2 binds directly to PY motifs (PPxY) in the C-termini of ENaC subunits via its WW domains, catalyzing channel polyubiquitination and subsequent clathrin-dependent endocytosis and degradation (25, 26). This Nedd4-2/ENaC interaction is dynamically regulated by aldosterone-induced SGK1, which phosphorylates Nedd4-2 at Ser444 and Ser338 (27). Phosphorylation promotes 14-3-3 protein binding to Nedd4-2, disrupting its interaction with ENaC and increasing Na^+^ reabsorption (28, 29). While hormonal regulation of ENaC, particularly via aldosterone signaling, is well characterized, there remain incompletely understood aspects of the post-translational mechanisms that anchor active channels at the apical membrane. Casein kinase 2 (CK2) is a constitutively active, highly conserved serine/threonine kinase that exists as a tetrameric holoenzyme (30, 31). Beyond its established roles in cell proliferation, apoptosis, DNA repair, and circadian rhythm regulation, CK2 has emerged as an important regulator of ion-channel function (32). CK2 has been shown to phosphorylate voltage-gated sodium channels (Nav) and voltage-gated potassium channels (KCNQ) within conserved cytoplasmic “anchor” motifs, and this phosphorylation functions as a molecular switch that enhances ankyrin-G (also known as ankyrin-3, encoded by the ANK3 gene) binding affinity (33, 34). Ankyrin-G, in turn, functions as a scaffolding protein that links phosphorylated channels to the cortical actin/spectrin-based membrane cytoskeleton, thereby promoting their proper trafficking, localization, and retention at specialized membrane domains (35, 36). Notably, the C-terminus of β-ENaC contains CK2 phosphorylation sites within a conserved anchor motif structurally analogous to those present in Nav and KCNQ channels (37, 38). Although these channel families are evolutionarily unrelated, the established role of CK2-dependent phosphorylation in promoting ankyrin-G recruitment to Nav and KCNQ channels suggests a conserved regulatory mechanism. Interestingly, ankyrin-G has been shown to modulate ENaC activity in murine principal cells (37), and pharmacological CK2 inhibition is shown to suppress ENaC function and enhance natriuresis (38, 39). Thus, targeting ENaC activity or its regulatory mechanisms could offer new strategies for treating hypertension.

Despite these observations, the functional significance of CK2/Ank-3 in renal epithelium, and its consequences for Na^+^ homeostasis, remain incompletely identified at the molecular level. Here, we demonstrate that CK2-dependent phosphorylation of a conserved regulatory motif within the β-ENaC C-terminus is required for Ank-3 binding, which stabilizes ENaC at the apical membrane and is critical for channel activity and appropriate renal Na^+^ excretion. Using structure-guided mutagenesis combined with advanced live-cell imaging (TIRF-FRAP, FRET) and patch-clamp electrophysiology, we show that disruption of either the CK2 consensus site or the Ank-3 binding motif abolishes ENaC surface mobility and suppresses macroscopic current density. Principal cell-specific deletion of CK2 or Ank-3, or pharmacologic CK2 inhibition, eliminates ENaC activity in murine native collecting ducts and drives markedly accelerated urinary Na^+^ loss in vivo. These results identify CK2-dependent Ank-3 scaffolding as an essential post-translational mechanism for ENaC regulation, maintaining apical ENaC expression and renal Na^+^ reabsorption, with important implications for sodium homeostasis and hypertension.

## Results

### Casein kinase 2 phosphorylation and ankyrin-3 binding sites within the β-ENaC C-terminus form a regulatory anchor motif necessary for ENaC trafficking and activity

**Figure 1:**
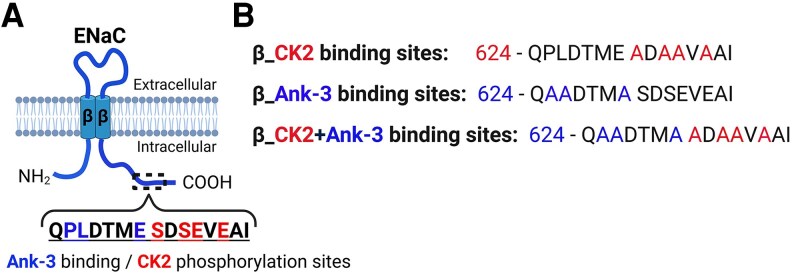
CK2-dependent phosphorylation and Ank-3 binding clusters in the β-ENaC C-terminal region. A) Schematic of the β-ENaC subunit showing the C-terminal region containing Ank-3 binding sites (blue) and CK2 phosphorylation sites (red), indicated by the dashed box. B) β-ENaC C-terminal sequences numbered according to the full-length protein, highlighting an Ank-3 binding cluster and a nearby CK2 phosphorylation cluster. Alanine substitutions introduced into the CK2 phosphorylation cluster beginning at residue 631 (β_CK2), the Ank-3 binding cluster beginning at residue 625 (β_Ank-3), or both overlapping clusters (β_CK2+Ank-3) are highlighted in red and blue, respectively.

Membrane expression of ENaC is dynamically regulated through post-translational modifications and scaffolding protein interactions (40–42). CK2 phosphorylates the β-ENaC-subunit at a consensus site in the distal C-terminus (43, 44), and we previously demonstrated that substitution of serine 631 (S631) with alanine abolishes CK2-dependent phosphorylation of β-ENaC (44). Here, we aimed to determine whether CK2 phosphorylation contributes to the formation of an Ank-3-dependent regulatory anchor motif. We mapped the residues within the distal intracellular C-terminus of β-ENaC predicted to work as CK2 phosphorylation or Ank-3 binding sites. A schematic of β-ENaC highlights candidate CK2 phosphorylation sites and Ank-3 binding sites located in close proximity, suggesting the presence of a composite regulatory anchor motif (Fig. 1A). To directly test the functional relevance of these residues, we used site-directed mutagenesis to selectively disrupt each region. This strategy generated β_CK2-site, β_Ank-3-site, and β_CK2+Ank-3-site constructs, in which the respective target residues were substituted with alanine (Fig. 1B). These mutant β-ENaC constructs provided a platform to define how CK2-mediated phosphorylation and Ank-3 binding regulate ENaC trafficking to the plasma membrane and channel activity.

**Figure 2:**
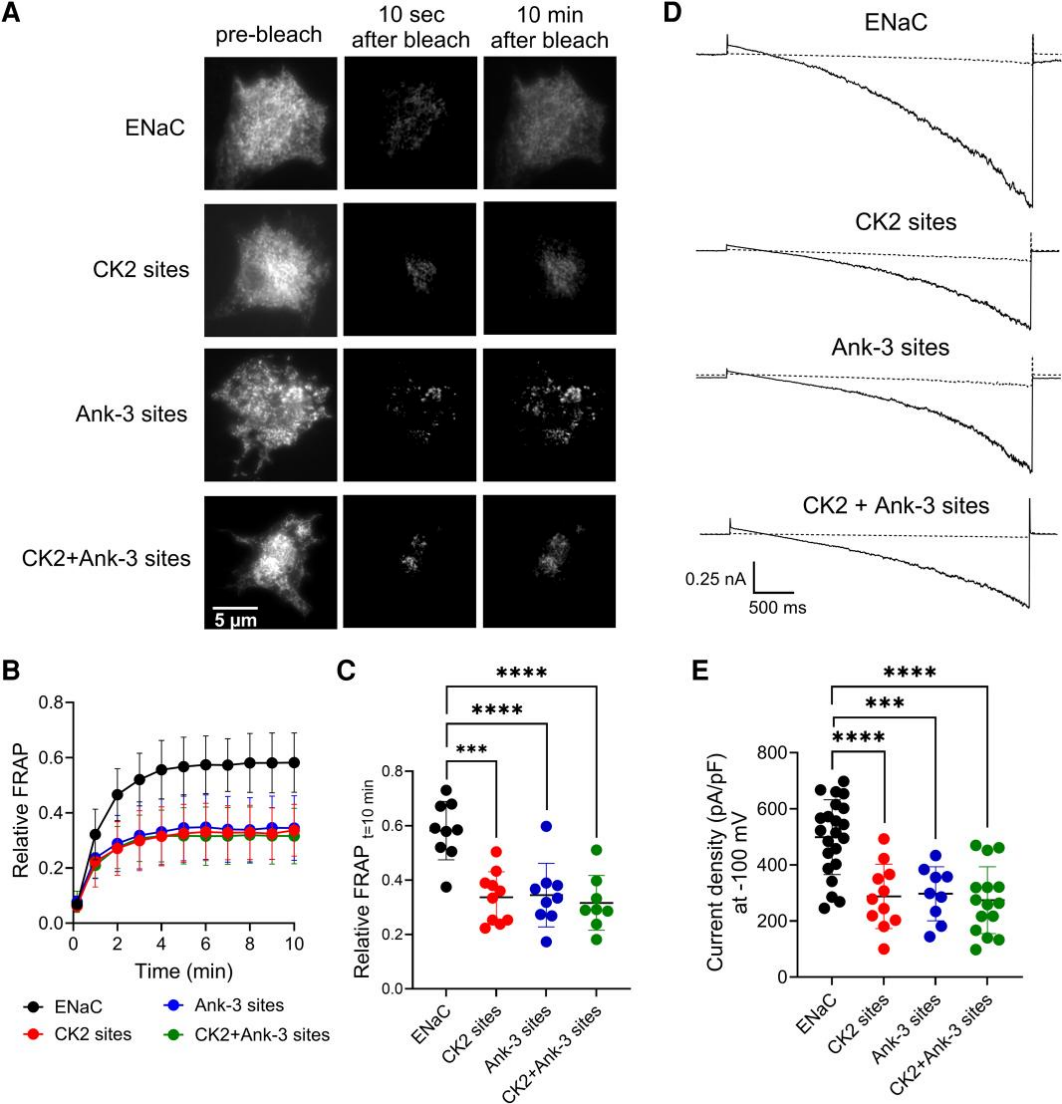
Phosphorylation of β-ENaC by CK2 within the anchoring motif is required for Ank-3-dependent regulation of channel trafficking and activity. A) Representative TIRF-FRAP images of COS-7 cells expressing eYFP-ENaC variants, including WT β-ENaC (top row), the CK2-site mutant (second row), the Ank-3-site mutant (third row), and the double-cluster mutant (fourth row). Images show prebleach, 10 s after bleach, and 10 min after bleach. B) Time-dependent FRAP curves at the plasma membrane for cells expressing WT eYFP-ENaC (black), the CK2-site mutant (red), the Ank-3-site mutant (blue), and the CK2+Ank-3 double mutant (green). Data represent mean recovery values from 8 to 10 cells obtained across 2–3 independent transfections corresponding to the imaging conditions shown in (A). C) Summary of FRAP values at 10 min after bleach for cells expressing WT and mutant eYFP-ENaC constructs. Individual data points are shown for WT (black), CK2-site mutants (red), Ank-3-site mutants (blue), and CK2+Ank-3 double mutants (green). Data are from the same experiments as in (A). D) Representative whole-cell current recordings from CHO cells expressing WT mENaC, the CK2-site mutant, the Ank-3-site mutant, or the CK2+Ank-3 double mutant. Traces show currents measured before and after application of 10 µM amiloride (dotted lines). E) Quantification of amiloride-sensitive ENaC current density (pA/pF) measured at −100 mV (plotted as positive values) in whole-cell voltage-clamped CHO cells expressing WT (black), CK2-site mutant (red), Ank-3-site mutant (blue), or CK2+Ank-3 double-mutant (green) channels. Each point represents an individual cell, with mean ± SEM indicated. Data are obtained from 9 to 22 cells across 3–5 independent transfections and correspond to the recordings shown in (D). Statistical significance for all data was determined using 2-tailed unpaired Student's t test (***P < 0.001; ****P < 0.0001).

To test whether CK2 phosphorylation within the β-ENaC anchor motif is required for Ank-3-dependent regulation of ENaC, we examined channel trafficking at the plasma membrane in COS-7 cells expressing wild-type (WT) or mutant β-ENaC constructs. We used total internal reflection fluorescence (TIRF) microscopy combined with fluorescence recovery after photobleaching (FRAP) imaging to monitor the dynamics of eYFP-tagged ENaC at the cell surface. In cells expressing the WT ENaC construct, strong fluorescence was observed at the membrane before bleaching, and rapid recovery occurred within 10 s, with near-complete restoration by 10 min (Fig. 2A, top row). In contrast, ENaC constructs carrying mutations in the CK2 site, the Ank-3 site, or both sites showed markedly impaired fluorescence recovery, exhibiting minimal recovery at 10 s and remaining strongly reduced at 10 min postbleach (Fig. 2A, rows 2–4). Time-course analysis of relative FRAP at the plasma membrane over 10 min showed that WT ENaC recovered to 60% (*n* = 9) of initial fluorescence, whereas recovery was significantly reduced to 25–30% in the β_CK2-site mutant (*n* = 10), the β_Ank-3-site mutant (*n* = 9), and the β_CK2+Ank-3 combined mutant (*n* = 8) (Fig. 2B and C). These results demonstrate that phosphorylation of CK2 sites and interaction with Ank-3 are both required to maintain ENaC mobility and trafficking at the plasma membrane. To confirm that the effects of CK2 inhibition were specific to the CK2 phosphorylation sites within the β-ENaC anchor motif, we tested whether the specific CK2 inhibitor TBB (4,5,6,7-tetrabromobenzotriazole) altered trafficking of ENaC constructs in which these sites were already disrupted. COS-7 cells were transfected with β-ENaC mutants at the CK2 site, the Ank-3 site, or both. Cells were preincubated with 200 nM TBB for 30 min, and TIRF-FRAP was then performed. WT ENaC FRAP was strongly attenuated in the presence of TBB, reaching only 25–30% of initial fluorescence by 10 min, similar to ENaC constructs lacking the β_CK2, β_Ank-3, or CK2+Ank-3 sites, which each reached only 25–30% of baseline over the same 10-min time course (Fig. S1A and B). Quantification of FRAP recovery at 10 min confirmed no significant differences among WT+TBB, β_CK2+TBB, β_Ank-3+TBB, or β_CK2+Ank-3+TBB (Fig. S1C). These results demonstrate that CK2 inhibition reduces WT ENaC trafficking to levels observed in the mutant constructs, with no additional effect in the CK2-site, Ank-3-site, or combined-site mutants.

To assess the functional consequence of impaired trafficking caused by disruption of the CK2 and Ank-3 sites, we measured whole-cell ENaC currents in CHO cells expressing WT or mutant β-ENaC. Cells expressing WT ENaC exhibited large inward amiloride-sensitive Na^+^ currents consistent with efficient surface expression, whereas mutation of the CK2 site, the Ank-3 site, or both resulted in a significant reduction in current density (Fig. 2D). At −100 mV, the current density decreased from 499.27 ± 44.47 pA/pF (*n* = 22) in WT to 287.57 ± 34.56 pA/pF (*n* = 11), 297.07 ± 32.06 pA/pF (*n* = 9), and 274.05 ± 30.73 pA/pF (*n* = 15) in the β_CK2, β_Ank-3, and β_CK2+Ank-3 mutants, respectively (Fig. 2E). These data demonstrate that CK2 phosphorylation and Ank-3 binding within the anchor motif are required not only to maintain ENaC trafficking at the membrane but also to sustain normal channel activity. We next directly tested whether pharmacological inhibition of CK2 by TBB alters macroscopic ENaC currents. Whole-cell patch-clamp analysis demonstrated that TBB treatment reduced WT ENaC currents to the same low levels observed in the β_CK2, β_Ank-3, and β_CK2+Ank-3 mutants (Fig. S1D). Quantification of current density at −100 mV confirmed that there were no significant differences between WT+TBB and any of the mutant constructs in the presence of TBB (Fig. S1E). Thus, similar to the FRAP data, blocking CK2 activity abolished the difference between WT and mutant ENaC, indicating that CK2-dependent phosphorylation within the anchor motif is required to maintain the enhanced current carried by WT ENaC.

### Ank-3 binding to β-ENaC requires CK2-dependent phosphorylation within an anchor motif

CK2 is known to phosphorylate ion channels such as NaV and KCNQ within a defined cytosolic ankyrin-binding “anchor” motif, where phosphorylation functions as a molecular switch that enables Ank-3 binding and regulates channel trafficking (45, 46). The CK2 phosphorylation site mapped within the C-terminus of β-ENaC resides in an analogous anchor motif, suggesting that ENaC may use the same CK2-dependent mechanism to recruit Ank-3 (37, 44). We therefore asked whether Ank-3 binds to the β-ENaC subunit, and if so, whether CK2 influences this interaction. To validate the specificity and reliability of our fluorescence resonance energy transfer (FRET) approach, we first performed proof-of-principle experiments in COS-7 cells using established positive and negative controls. Cells expressing the mCGY protein (a fusion protein containing eCFP and eYFP separated by 6 Gly residues) exhibited a strong increase in eCFP fluorescence upon eYFP photobleaching, indicating robust energy transfer (Fig. S2A, top row). In contrast, coexpressing of monomeric Yellow Fluorescent Protein with eCFP-Ank-3, eYFP-βENaC with monomeric Cyan Fluorescent Protein (mCFP), or eYFP-mt βENaC with mCFP showed no significant FRET response, indicating minimal nonspecific interaction (Fig. S2A, rows 2–4). Quantitative analysis confirmed these findings: FRET efficiency in the mCGY positive control was significantly higher (25–30%) compared with all negative controls, which remained at background levels (2–5%; Fig. S2B). These data validate the sensitivity and specificity of the FRET assay, providing a robust methodological foundation for subsequent experiments assessing Ank-3 binding to β-ENaC.

**Figure 3:**
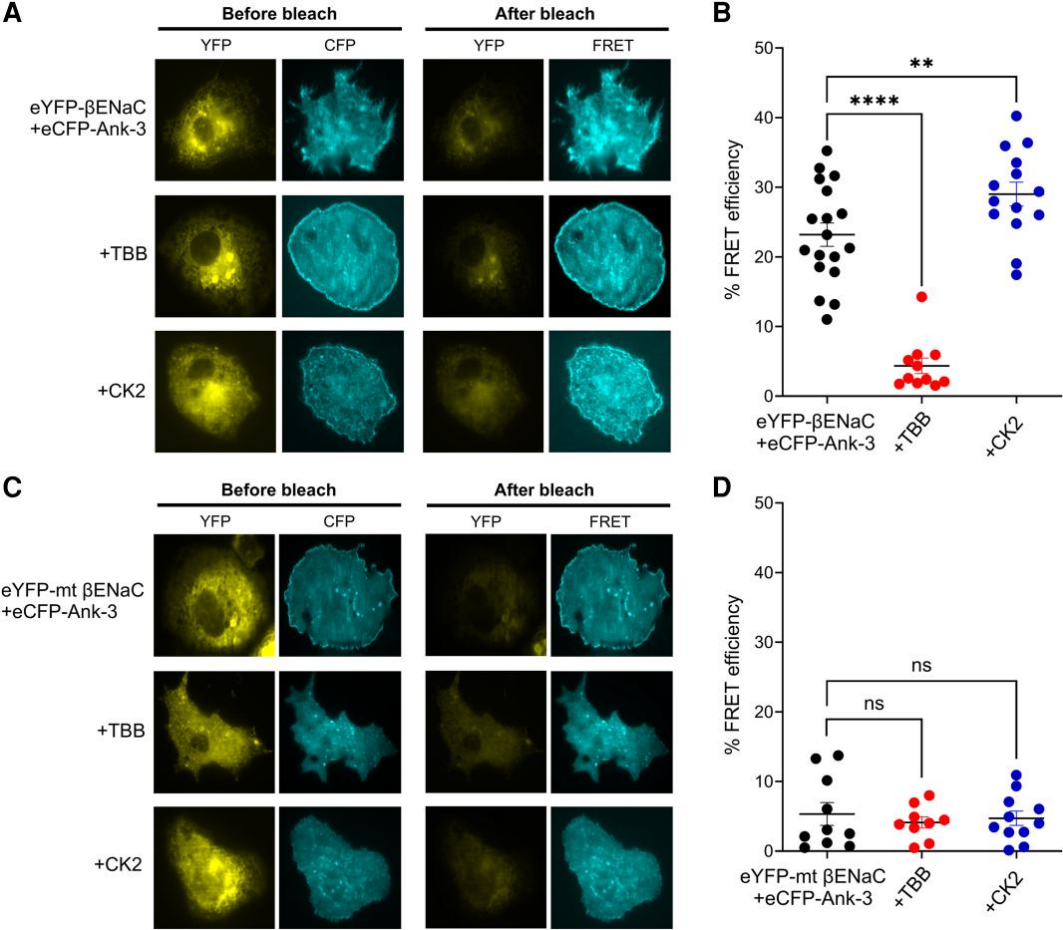
Ank-3 binds to β-ENaC subunit, and if so, whether CK2 influences this binding. A) Representative FRET images from COS-7 cells coexpressing eYFP-βENaC and eCFP-Ank-3. Left panels show YFP and CFP fluorescence before bleaching; right panels show YFP and FRET images after bleaching. Top row: untreated cells. Middle row: cells treated with the CK2 inhibitor TBB. Bottom row: cells cotransfected with CK2. B) Quantification of FRET efficiency for cells expressing eYFP-βENaC and eCFP-Ank-3 under the conditions shown in (A): untreated (black), TBB-treated (red), and CK2-overexpressing cells (blue). Data are from 11 to 20 cells and are presented as mean ± SEM. C) FRET imaging of COS-7 cells coexpressing the βENaC mutant lacking CK2/Ank-3 phosphorylation and binding sites (eYFP-mt βENaC) together with eCFP-Ank-3. Cells are shown under three conditions: untreated (top row), TBB treatment (middle row), and CK2 overexpression (bottom row). YFP and CFP channels are displayed before (left columns) and after (right columns) YFP bleaching. D) Quantification of FRET efficiency for COS-7 cells expressing eYFP-mt βENaC together with eCFP-Ank-3 under the conditions shown in (C): untreated (black), TBB-treated (red), and CK2-overexpressing cells (blue). Data are from 9 to 11 cells and are presented as mean ± SEM. Statistical significance for all data was determined using 2-tailed unpaired Student's t test (ns, *P* ≥ 0.05; **P < 0.01; ****P < 0.0001).

We then examined how CK2 activity alters β-ENaC/Ank-3 association using this validated FRET assay. In COS-7 cells coexpressing eYFP-tagged WT β-ENaC and eCFP-tagged WT Ank-3, photobleaching of eYFP resulted in a robust increase in eCFP fluorescence (Fig. 3A, top row). Inhibition of CK2 with TBB significantly reduced this FRET response, indicating that CK2 activity is necessary to promote Ank-3 binding (Fig. 3A, middle row). Moreover, overexpression of CK2 enhanced the FRET signal, further supporting the conclusion that CK2 phosphorylation strengthens the interaction between Ank-3 and β-ENaC (Fig. 3A, bottom row). Quantitative analysis of FRET efficiency showed that eYFP-βENaC and eCFP-Ank-3 exhibited ∼23% FRET efficiency, whereas CK2 inhibition with TBB reduced FRET efficiency to 5%, and CK2 overexpression increased FRET efficiency to 30% (Fig. 3B). These results demonstrate that β-ENaC and Ank-3 are in close molecular proximity and that CK2-dependent phosphorylation strengthens their interaction.

To directly test whether Ank-3 binding requires the defined Ank-3 sites within β-ENaC, we repeated the FRET experiments using a mutant β-ENaC construct lacking the Ank-3 binding sites (mt β-ENaC). In cells coexpressing eYFP-mt βENaC and eCFP-Ank-3, photobleaching of eYFP produced only minimal changes in eCFP fluorescence, indicating little or no interaction between Ank-3 and the mutant β-ENaC construct (Fig. 3C, top row). Importantly, neither inhibition of CK2 with TBB nor overexpression of CK2 altered this response (Fig. 3C, middle and bottom rows). Quantitative analysis confirmed that FRET efficiencies remained low and were not significantly different across all three conditions (at 5%; Fig. 3D). Thus, elimination of the Ank-3 sites in β-ENaC disrupts the ability of CK2 to promote Ank-3 binding, demonstrating that these residues are essential for forming the phosphorylation-dependent anchor motif.

### CK2 and ank-3 expressions in collecting duct principal cells are required to sustain ENaC activity in vivo

**Figure 4:**
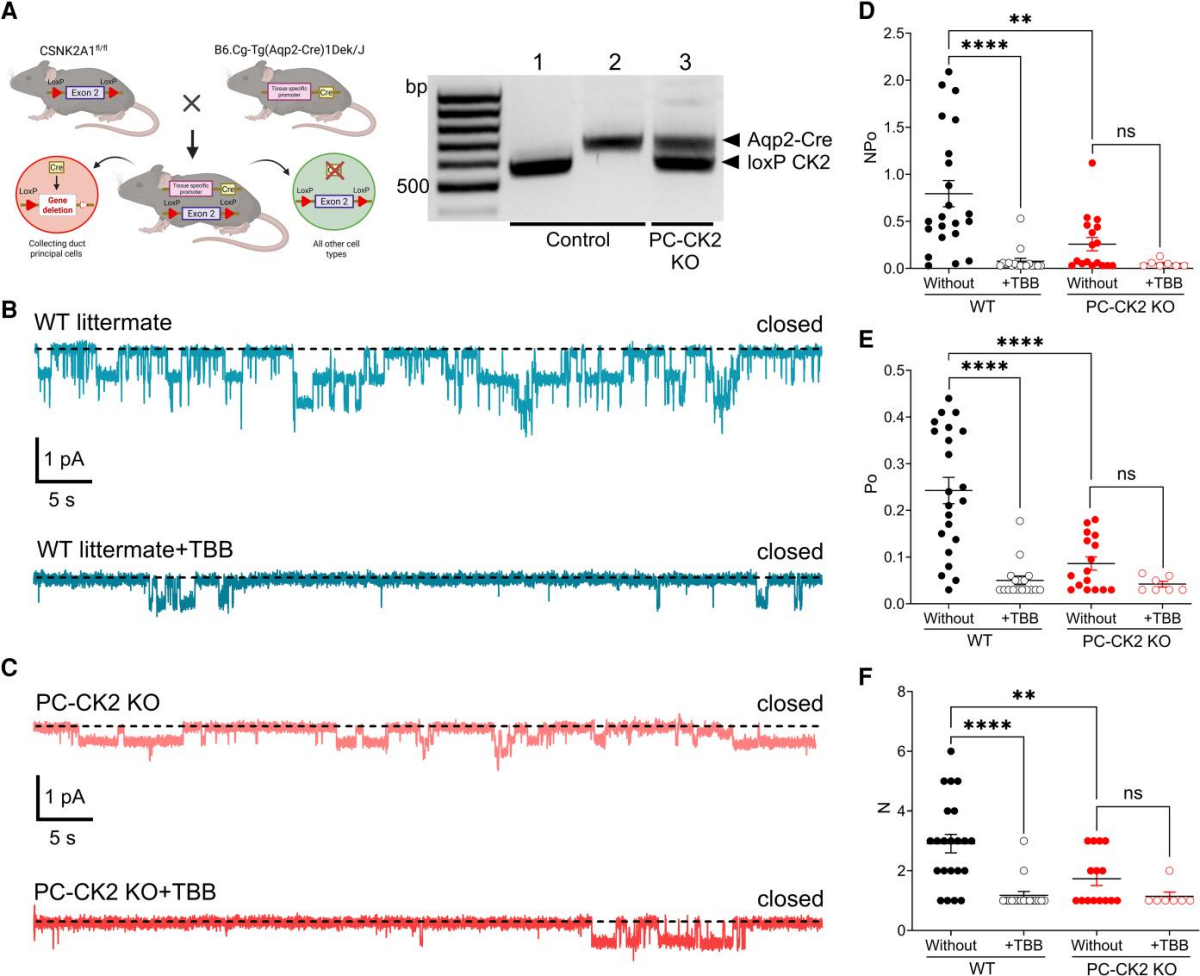
Expression of CK2 in principal cells is necessary for ENaC activity in the mouse collecting duct. A) Left, schematic of the breeding strategy used to generate PC-CK2 KO mice by crossing CSNK2A1^fl/fl^ animals with Aqp2-Cre transgenic mice. Right, representative gel containing PCR products from genotyping reactions for breeders (lanes 1 and 2) and PC-specific CK2 KO mice (lane 3). Bands corresponding to the *Aqp2-Cre* transgene and the floxed CK2 allele are indicated with arrowheads. The gel image was inverted (black to white) to enhance visual clarity without altering the underlying data. bp, base pairs. B, C) Representative gap-free ENaC single-channel current recordings obtained in the cell-attached configuration from the apical membrane of principal cells in freshly isolated collecting duct tubules from WT littermates and PC-CK2 KO mice. Traces are shown under baseline conditions (top) and after pretreatment of isolated tubules with TBB (200 nM, 30 min; bottom). Inward Na^+^ current is downwards with dashed lines indicating the closed state. D–F) Summary graph of ENaC activity (NPo), open probability (Po), and channel number (N) obtained from cell-attached recordings of WT littermate tubules and PC-CK2 KO tubules under baseline conditions (black and red filled circles) and after TBB pretreatment (open black and red circles). Measurements represent 7–22 patches collected from 3 to 4 mice, corresponding to the recordings shown in (B) and (C). Both male and female mice were used in similar proportions. Statistical significance for all data was determined using two-tailed unpaired Student's t test (ns, *P* ≥ 0.05; **P < 0.01; ****P < 0.0001).

CK2 and Ank-3 regulate ENaC trafficking to the plasma membrane and support channel activity in vitro, therefore we asked whether disrupting CK2 or Ank-3 expression specifically in principal cells would affect ENaC activity and dependent sodium excretion. To directly test this, we generated conditional knockout mouse models in which CK2 or Ank-3 were selectively deleted in principal cells of the collecting duct. CK2 is a tetramer kinase comprised of two catalytic α subunits and two regulatory β subunits. Mammals express three CK2 genes, two α subunit genes (CSNK2A1 and CSNK2A2) and one β subunit (CSNK2B). The predominant α subunit in the kidney and collecting duct is encoded by CSNK2A1, while CSNK2A2 (encoding α’) shows more restricted expression (47–50). CK2 phosphorylation sites are present in the binding motifs of several ankyrin-interacting proteins, suggesting functional coordination between these two protein families. The mammalian genome encodes three ankyrin genes (ANK1, ANK2, and ANK3). In the kidney, ANK2 and ANK3 are robustly expressed, whereas ANK1 shows minimal expression (51). Within epithelial tissues, these ankyrins have distinct subcellular localizations and functions. Ankyrin-3 typically functions at the junctional–lateral domain, where it coordinates the sorting of membrane proteins before they enter the apical membrane. In contrast, ankyrin-2 is more commonly associated with subapical endosomal trafficking and targeting intracellular organelles (52–57). Based on sequence homology within the ENaC C-terminal “anchor” motif, there is little priori indication that the channel would preferentially interact with Ank-3 over Ank-2. Nonetheless, Ank-3 regulates other ion channels, including NaV and KCNQ, through anchor motifs containing CK2-sensitive sites (45, 58). Ank-3 expression is responsive to aldosterone, a key hormonal regulator of ENaC function (59). Genotyping confirmed the presence of the Aqp2-cre transgene, which drives recombination specifically in principal cells (60, 61), along with floxed alleles of CK2 or Ank-3, verifying successful generation of principal cell-specific CK2 KO (PC-CK2 KO; Fig. 4A) and principal cell-specific Ank-3 knockout (PC-Ank-3 KO) mice (Fig. S3A). Both knockout lines appeared normal, with no significant differences in external phenotype, body weight, or general activity compared with WT littermates. Additionally, no significant differences were observed between male and female mice of either genotype. Both sexes were used in equal proportions across all experiments. These models provide the basis for in vivo experiments to test whether CK2 phosphorylation and Ank-3 binding regulate ENaC activity in the native renal epithelium.

To test whether CK2 is required for ENaC activity in vivo, we performed cell-attached patch-clamp recordings from the apical membrane of principal cells in freshly isolated collecting ducts from WT littermates and PC-CK2 KO mice. In WT tubules, robust inward Na^+^ currents were detected under baseline conditions (Fig. 4B, top). However, pretreatment with the CK2 inhibitor TBB (200 nM, 30 min) substantially reduced ENaC activity in WT cells (Fig. 4B, bottom). Additionally, principal cells from PC-CK2 KO mice exhibited markedly reduced basal ENaC activity (Fig. 4C, top), and TBB treatment did not further suppress ENaC activity (Fig. 4C, bottom). ENaC activity, recorded as NP_0_, was significantly reduced in TBB-pretreated WT mice compared with untreated WT controls, decreasing from 0.79 ± 0.14 to 0.08 ± 0.03 (Fig. 4D). This loss of activity reflected reductions in both open probability (P_0_), which decreased from 0.24 ± 0.03 to 0.05 ± 0.009, and the number of active channels per patch (N), which decreased from 2.9 ± 0.31 to 1.18 ± 0.13 (Fig. 4E and F). Consistent with the magnitude of suppression observed in TBB-treated WT tubules, principal cells from PC-CK2 KO mice exhibited substantially reduced baseline ENaC activity relative to littermate controls, and NP_0_, P_0_, and N values in the knockout were not further decreased by TBB, indicating that CK2 deletion eliminates the TBB-sensitive component of ENaC regulation (Fig. 4D–F). These data demonstrate that CK2 expression in principal cells is necessary to maintain full ENaC activity in the native collecting duct, and the absence of further inhibition by TBB in PC-CK2 KO mice confirms that this regulation is specifically attributable to loss of CK2 function.

Given that CK2 deletion eliminates the ENaC activity in vivo, and that Ank-3 is the downstream binding partner engaged by CK2 within the β-ENaC anchor motif, we next asked whether loss of Ank-3 in principal cells would give the same physiological phenotype. To do this, we performed cell-attached patch-clamp recordings from the apical membrane of principal cells in collecting ducts isolated from WT and PC-Ank-3 KO mice. Ank-3 deletion markedly diminished ENaC activity in the native collecting duct. In the PC-Ank-3 KO mice, ENaC currents were minimal under basal conditions, reaching the same low level observed in WT tubules only after CK2 inhibition with TBB (Fig. S3B and C). TBB treatment failed to further suppress ENaC activity in the Ank-3 KO, indicating that Ank-3 loss blocks the CK2-dependent regulatory step. ENaC activity (NP_0_) was significantly reduced in PC-Ank-3 KO mice compared with WT littermates, with NP_0_ decreasing from 0.72 ± 0.20 in WT to 0.27 ± 0.09 in PC-Ank-3 KO, and this reduction reflected decreases in both channel open probability P_0_ which declined from 0.22 ± 0.04 in WT to 0.11 ± 0.02 in PC-Ank-3 KO, and channel number N which decreased from 2.5 ± 0.43 in WT to 1.59 ± 0.19 in PC-Ank-3 KO (Fig. S3D–F). These data show that Ank-3 expression in principal cells is required for full ENaC activity in vivo and support a model in which Ank-3 is a necessary effector of CK2-dependent anchoring within the β-ENaC regulatory motif.

### Deletion of CK2 or Ank-3 in principal cells accelerates renal sodium excretion in vivo under physiological conditions

**Figure 5:**
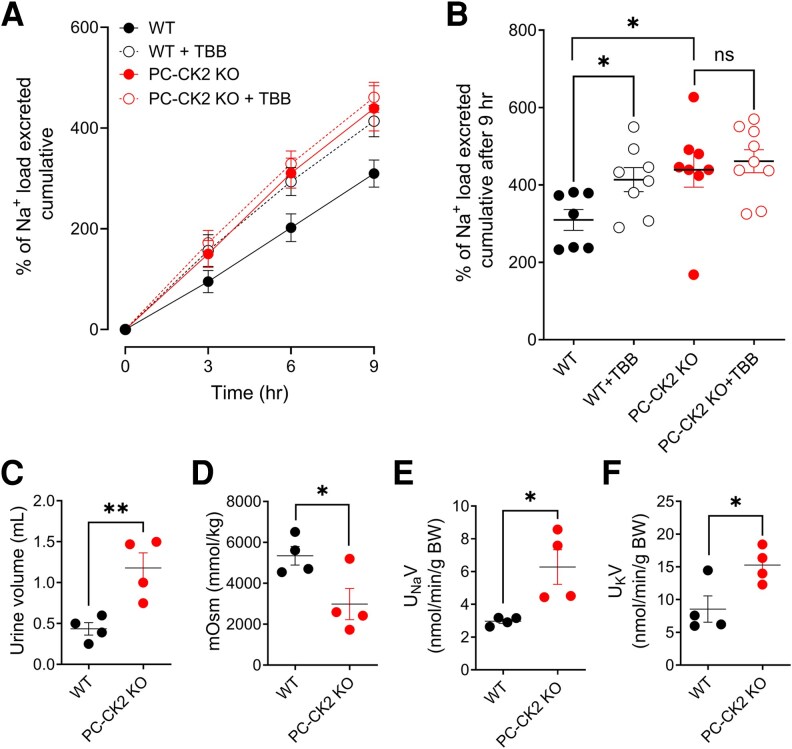
Principal-cell CK2 deficiency alters systemic sodium excretion and urinary electrolyte composition. A) Cumulative Na^+^ excretion over a 9-h period in WT mice (black filled circles, *n* = 7), WT mice treated with TBB (open black circles, *n* = 8), PC-CK2 KO mice (red filled circles, *n* = 8), and PC-CK2 KO mice treated with TBB (open red circles, *n* = 9). Mice received an intraperitoneal Na^+^ load (100 µL, 0.9% NaCl) at time 0, and cumulative excretion was measured every 3 h. B) Cumulative Na^+^ excretion at the 9-h endpoint for WT and PC-CK2 KO mice with or without TBB pretreatment. Data are plotted as mean ± SEM. C–F) Summary graphs showing 24 h urine volume (mL), Osmolality (mmol/kg), urinary Na^+^ excretion (U_Na_V), and urinary K^+^ excretion (U_K_V) for WT littermate (black circles, *n* = 4) and PC-specific CK2 KO mice (red squares, *n* = 4). Data are displayed as mean ± SEM. Both male and female mice were used in similar proportions. Statistical significance for all data was assessed using two-tailed unpaired Student's t tests (ns, *P* ≥ 0.05; *P < 0.05).

To determine the physiological relevance of CK2- and Ank-3-dependent ENaC regulation, we examined renal sodium excretion in PC-CK2 KO and PC-Ank-3 KO mice following an injection of Na^+^ load. In WT mice, cumulative Na^+^ excretion increased progressively over time and was enhanced by pretreatment with the CK2 inhibitor TBB (Fig. 5A). PC-CK2 KO mice exhibited significantly greater cumulative Na^+^ excretion than WT littermates, and TBB did not further augment sodium excretion in these knockout animals. At 9 h after sodium loading, cumulative sodium excretion in WT mice was 310 ± 27% of the administered sodium load, and was increased to 414 ± 31% by TBB pretreatment, whereas in PC-CK2 KO mice cumulative sodium excretion was significantly higher at 439 ± 45%, and TBB did not further increase sodium excretion in the KO animals, which remained at 461 ± 30% (Fig. 5B). Metabolic balance studies confirmed impaired ENaC-dependent sodium reabsorption in PC-CK2 KO mice. Compared with WT littermates, PC-CK2 KO mice exhibited a significant increase in 24-hr urine volume, rising from 0.44 ± 0.07 mL in WT to 1.18 ± 0.18 mL in PC-CK2 KO animals (Fig. 5C). Urine osmolality was significantly reduced, decreasing from 5,342 ± 455 mmol/kg in WT to 2,985 ± 761 mmol/kg in PC-CK2 KO mice (Fig. 5D). Moreover, urinary sodium excretion (U_Na_V) was elevated, increasing from 2.97 ± 0.13 nmol/min/g BW in WT to 6.28 ± 1.06 nmol/min/g BW in PC-CK2 KO mice (Fig. 5E). Urinary potassium excretion (U_K_V) was significantly increased in PC-CK2 KO animals (Fig. 5F). These metabolic parameters demonstrate a clear natriuretic phenotype in the absence of PC-CK2 and are consistent with impaired ENaC-mediated sodium reabsorption.

Similarly, deletion of Ank-3 in principal cells also accelerated renal sodium excretion in vivo. PC-Ank-3 KO mice displayed significantly greater cumulative sodium excretion compared with WT controls after sodium loading, and TBB treatment did not produce any additional increase in Na^+^ excretion in the knockout animals (Fig. S4A). At 9 h, cumulative Na^+^ excretion in WT mice was 350 ± 28% of the administered sodium load, increased to 565 ± 28% with TBB pretreatment, whereas PC-Ank-3 KO mice exhibited significantly higher cumulative Na^+^ excretion at 531 ± 63%, with no further increase following TBB (518 ± 88%; Fig. S4B). Metabolic balance testing further demonstrated altered urine composition in PC-Ank-3 KO mice. Compared with WT littermates, PC-Ank-3 KO animals exhibited significantly increased 24-h urine volume, rising from 0.57 ± 0.06 mL in WT to 1.36 ± 0.11 mL in PC-Ank-3 KO mice (Fig. S4C). In addition, urine osmolality was significantly reduced in PC-Ank-3 KO mice (Fig. S4D). Consistent with impaired distal Na^+^ reclamation, U_Na_V was significantly elevated in PC-Ank-3 KO animals (Fig. S4E), and U_K_V was also significantly increased in PC-Ank-3 KO animals (Fig. S4F). These findings indicate that Ank-3 deletion disrupts normal ENaC-dependent Na^+^ handling, leading to increased Na^+^ loss and reduced urinary concentration ability, similar to the phenotype observed in PC-CK2 KO mice.

Baseline urinary parameters were assessed from 3-h urine collections in WT, PC-CK2 KO, and PC-Ank-3 KO mice maintained on standard chow. Under these unchallenged conditions, both KO lines showed increase urine output than WT littermates (0.383 ± 0.019 mL in PC-CK2 KO and 0.401 ± 0.025 mL in PC-Ank-3 KO vs. 0.311 ± 0.021 mL in WT), accompanied by modestly higher U_Na_V (1.142 ± 0.038 and 1.151 ± 0.048 vs. 1.103 ± 0.047 nmol/min/g BW, respectively) and U_K_V (3.517 ± 0.102 and 3.432 ± 0.086 vs. 3.209 ± 0.094 nmol/min/g BW), consistent with a constitutive deficit in ENaC-mediated Na^+^ reabsorption (Table S1). Baseline blood electrolytes and plasma aldosterone were obtained from the same animals. Serum creatinine (0.241 ± 0.057, 0.274 ± 0.041, and 0.259 ± 0.037 mg/dL for WT, PC-CK2 KO, and PC-Ank-3 KO, respectively), cystatin C (0.967 ± 0.081, 1.03 ± 0.104, and 0.972 ± 0.098 mg/L), and blood urea nitrogen (BUN) (27.1 ± 0.95, 30.4 ± 1.01, and 26.8 ± 0.98 mg/dL) were within the normal range and did not differ significantly between genotypes, confirming intact glomerular filtration. Plasma Na^+^ trended lower across genotypes (161.4 ± 5.04 mmol/L in WT vs. 157.8 ± 3.89 in PC-CK2 KO and 152.6 ± 7.54 in PC-Ank-3 KO), with corresponding modest increases in plasma Cl^−^ (114.8 ± 3.48 vs. 119.2 ± 5.03 and 121.7 ± 8.46 mmol/L), while plasma K^+^ remained comparable across groups (3.7 ± 0.102, 4.1 ± 0.097, and 3.9 ± 0.114 mmol/L). Most notably, plasma aldosterone was approximately doubled in both KO lines relative to WT (409.1 ± 23.31 and 414.3 ± 22.53 vs. 232 ± 18.16 pg/mL), supporting compensatory RAAS activation in response to ongoing renal Na^+^ loss (Table S1).

**Figure 6:**
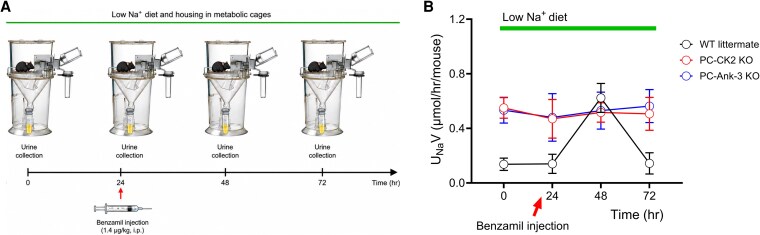
Loss of CK2 or Ank-3 abolishes benzamil-sensitive Na^+^ reabsorption. A) Experimental protocol. WT littermate, PC-CK2 KO, and PC-Ank-3 KO mice were placed in metabolic cages and maintained on a low-Na^+^ diet. Urine samples were collected at 0, 24, 48, and 72 h. Benzamil (1.4 mg/kg, i.p.) was administered at 24 h. B) U_Na_V during low-Na^+^ diet in WT littermate, PC-CK2 KO, and PC-Ank-3 KO mice. Benzamil (1.4 mg/kg, i.p.) was administered at 24 h (red arrow). Data are presented as mean ± SEM.

To further probe ENaC function under conditions of Na^+^ conservation, we examined the natriuretic response to acute ENaC blockade with benzamil, a selective amiloride analog. Mice were housed in metabolic cages and maintained on a Na^+^-deficient diet (<0.01%), with urine collected at 0, 24, 48, and 72 h and benzamil (1.4 mg/kg, i.p.) administered at the 24 h time point (Fig. 6A). Under sodium restriction, WT mice appropriately conserved Na^+^, maintaining very low baseline U_Na_V (0.14 ± 0.04 µmol/h/mouse at 0 and 24 h). Benzamil provoked a pronounced natriuresis in these animals, with U_Na_V rising roughly 4-fold to a peak of 0.62 ± 0.08 µmol/h/mouse at the 48 h collection before returning to baseline (0.14 ± 0.06 µmol/h/mouse) by 72 h, reflecting blockade of the ENaC that normally reabsorbs Na^+^ under these conditions (Fig. 6B). In contrast, PC-CK2 KO and PC-Ank-3 KO mice already exhibited elevated U_Na_V during Na^+^ restriction (0.55 ± 0.06–0.53 ± 0.08 µmol/h/mouse at baseline) that exceeded the conserving WT level, and benzamil produced little or no further increase, with U_Na_V remaining essentially flat (0.52 ± 0.06–0.53 ± 0.09 µmol/h/mouse) across all four time points. The benzamil-induced change in Na^+^ excretion (ΔU_Na_V) was therefore large in WT mice but minimal in the KO lines, consistent with markedly reduced ENaC activity and confirming that the natriuretic phenotype reflects loss of functional ENaC.

These findings demonstrate that principal-cell CK2 and Ank-3 are each required for normal ENaC-dependent sodium handling in vivo. Loss of either component produces concordant natriuretic phenotype, accelerated Na^+^ excretion, increased urine volume, and reduced urinary concentration.

## Discussion

**Figure 7:**
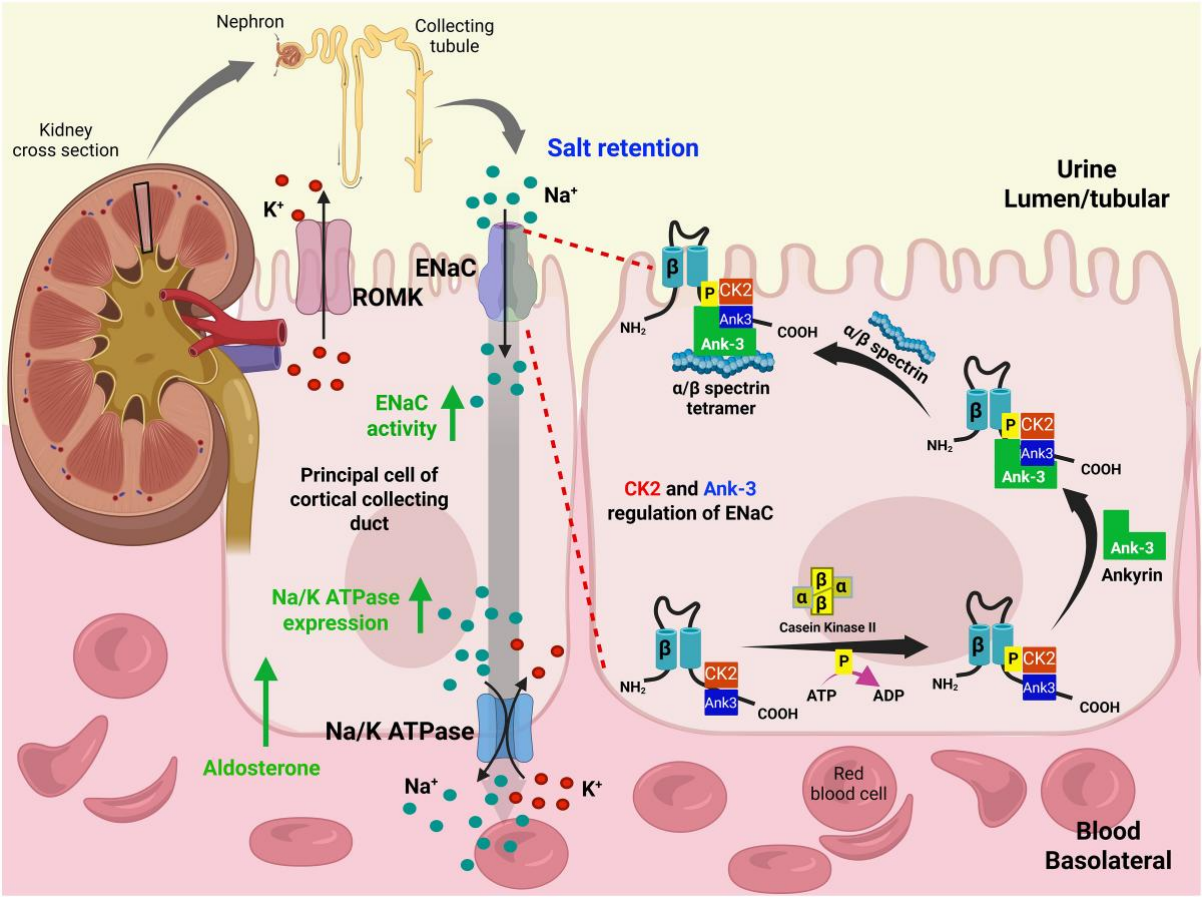
CK2- and Ankyrin-3–dependent regulation of ENaC in principal cells of the cortical collecting duct. CK2 phosphorylates on a regulatory cluster within the β-ENaC C-terminal domain, comprising a CK2-associated cluster (red) positioned adjacent to an Ank-3-interacting cluster (blue), thereby enabling effective association with Ank-3. This interaction is thought to link ENaC to the ankyrin-spectrin cytoskeletal scaffold, promoting efficient trafficking and stable retention of the channel at the apical membrane. Elevated ENaC abundance and activity at the luminal surface facilitate Na^+^ entry, which is complemented by Na^+^ extrusion through the basolateral Na^+^/K^+^-ATPase, thereby sustaining transepithelial Na^+^ transport and contributing to renal salt homeostasis.

The present study establishes CK2-dependent phosphorylation of β-ENaC and subsequent Ank-3 scaffolding as an essential post-translational mechanism that maintains ENaC at the apical membrane of principal cells and sustains renal Na^+^ reabsorption. CK2 modulates ion-channel function by phosphorylating scaffold proteins or directly phosphorylating channel subunits to influence their gating behavior (45, 62). Our findings demonstrate that CK2 phosphorylates conserved clusters of serine residues located mainly within the β-ENaC C-terminus to create a high-affinity Ank-3 binding site (Fig. 7). This phosphorylation-dependent interaction stabilizes ENaC at the apical membrane, supporting sustained channel presence and maintaining its basal open probability under physiological conditions. Disruption of this regulatory axis, through genetic deletion of CK2 or Ank-3 in principal cells, targeted mutation of the phosphorylation site or binding motifs, or pharmacological CK2 inhibition, consistently diminished ENaC activity, leading to increased urinary Na^+^ excretion and an impaired ability to retain sodium following a salt load.

Our FRET experiments directly demonstrate that CK2 activity dynamically regulates the strength of the β-ENaC/Ank-3 interaction. CK2 inhibition reduced FRET efficiency, while CK2 overexpression enhanced it, indicating that the phosphorylation state of β-ENaC determines Ank-3 binding affinity. Despite the evolutionary divergence of ENaC and Nav and KCNQ channel families, CK2-mediated phosphorylation of conserved motifs in Nav and KCNQ channels promotes their binding to ankyrin-G at the axon initial segment and nodes of Ranvier (34, 35, 63, 64). The structural similarity of the anchor motifs in β-ENaC, Nav channels, and KCNQ channels suggests that this represents a modular regulatory cassette that can be inserted into diverse channel scaffolds to confer CK2/ankyrin-dependent membrane stabilization. Furthermore, CK2-mediated phosphorylation of calmodulin strengthens its binding to KCNQ2 channels and stabilizes channel activity (65), demonstrating that CK2 can regulate channel function through both direct phosphorylation and modification of associated regulatory proteins.

The identification of adjacent CK2 and Ank-3 binding clusters within the β-ENaC C-terminus provides a molecular explanation for how phosphorylation controls scaffolding protein engagement. CK2 consensus sites typically conform to the sequence S/T-X-X-D/E, where the acidic residues at the +3 position are critical determinants of substrate recognition (30, 66). The β-ENaC C-terminus contains multiple serine residues flanked by glutamate residues that match this consensus, and our previous work demonstrated that S631 is a bona fide CK2 substrate (44). Phosphorylation at this position likely introduces negative charge that complements a positively charged patch within the ankyrin repeat domain, thereby strengthening the electrostatic interaction between channel and scaffold. This model is consistent with structural studies of ankyrin-G showing that its membrane-binding domain contains multiple positively charged surfaces that recognize negatively charged motifs in target proteins (67, 68). The proximity of the CK2 and Ank-3 sites within the β-ENaC suggests a composite motif in which phosphorylation and binding are functionally coupled.

The physiological importance of the CK2/Ank-3/ENaC axis is emphasized by the profound natriuretic phenotype observed in PC-CK2 KO and PC-Ank-3 KO mice. Both knockout models exhibited markedly reduced ENaC activity in native collecting ducts, accelerated UNaV following a salt load, increased 24-h urine volume, and reduced urine osmolality, findings consistent with impaired ENaC-mediated sodium reabsorption. The accompanying water loss is best understood as an osmotic consequence of the primary sodium-handling defect rather than a failure of AQP2-mediated water reabsorption. This coupling of reduced ENaC activity to the loss of both sodium and water is well established in models of distal nephron sodium-transport deficiency. Principal cell-specific mineralocorticoid receptor (MR) KO mice, in which reduced ENaC activity is confined to the collecting duct and late connecting tubule, grow normally on a standard diet but lose sodium and water and fail to maintain body weight when challenged with a low-sodium diet (69), a close parallel to the phenotype of our models. In the more severe case of global MR deletion, mice die within the second week of life from sodium and water loss. In both settings, water loss tracks the underlying natriuresis rather than a primary defect in AQP2, in agreement with our findings. Pharmacological CK2 inhibition with TBB did not further suppress ENaC activity or enhance natriuresis in either knockout model, demonstrating that the observed phenotypes result specifically from loss of CK2 or Ank-3 function rather than off-target effects and strongly supports a linear pathway in which CK2 acts upstream of Ank-3. The magnitude of sodium wasting observed in our knockout models is substantial, yet compensatory mechanisms, including increased renin–angiotensin–aldosterone system activity, enhanced proximal tubule reabsorption, and behavioral adaptations such as increased salt appetite and water intake, partially offset the collecting duct defect (70, 71).

Nonetheless, the increased urinary K^+^ excretion observed in the KO animals is unexpected, as classical models predict that diminished ENaC-mediated Na^+^ entry should reduce the electrochemical driving force for K^+^ secretion through ROMK (72). However, several mechanisms may explain this paradox. First, the increased distal Na^+^ and fluid delivery resulting from impaired ENaC-mediated reabsorption increases tubular flow rate, which is a potent stimulus for Ca^2+^-activated BK (maxi-K) channels that mediate flow-induced K^+^ secretion independently of ENaC (73, 74). We note that, in the intact CCD, a component of flow-stimulated net K^+^ secretion is ENaC-dependent, reflecting the lumen-negative transepithelial voltage generated by concurrent ENaC-mediated Na^+^ entry (75), however, flow also activates BK channels through a mechanotransduction-dependent rise in intracellular Ca^2+^ that does not require Na^+^ entry through ENaC. Second, the Na^+^ wasting caused by impaired ENaC function activates the renin–angiotensin–aldosterone system; consistent with this, plasma aldosterone was approximately doubled in both KO lines, providing an additional ENaC-independent stimulus for distal K^+^ secretion. Thus, under the conditions of our model, chronic loss of principal-cell ENaC accompanied by supraphysiologic distal flow and secondary hyperaldosteronism, these flow- and aldosterone-driven pathways can enhance K^+^ excretion even when ENaC-mediated Na^+^ reabsorption is reduced, consistent with reports that BK channel-dependent K^+^ excretion can be dissociated from ENaC-mediated Na^+^ reabsorption under conditions of high tubular flow (76). Importantly, the kaliuretic phenotype does not diminish the strength of evidence for impaired ENaC activity, as the natriuresis, reduced channel open probability, and decreased channel number are all internally consistent and directly demonstrate loss of ENaC function. The increased K^+^ excretion likely reflects a secondary consequence of altered tubular dynamics rather than a direct effect on K^+^ channels, consistent with the coupled nature of Na^+^ reabsorption and K^+^ secretion in principal cells.

This phenotype mirrors clinical observations in patients with pseudohypoaldosteronism type 1, who harbor loss-of-function mutations in ENaC and present with renal salt wasting, hyperkalemia, and metabolic acidosis (77). If CK2/Ank-3 coupling is required to maintain basal ENaC activity, then dysregulation of this pathway could contribute to inappropriate sodium retention and elevated blood pressure. Supporting this concept, in vivo studies demonstrated that CK2 inhibition reduces ENaC activity in the native collecting duct and elicits a robust natriuretic response comparable to amiloride, underscoring the essential role of CK2 in maintaining physiologic sodium reabsorption (39). Our data provide a molecular mechanism for these observations by demonstrating that CK2 inhibition disrupts Ank-3 binding, destabilizes ENaC at the membrane, and reduces channel activity. Conversely, enhanced CK2 activity or increased Ank-3 expression could pathologically increase ENaC function and drive hypertension. Consistent with this possibility, Ank-3 expression is upregulated by aldosterone, a hormone that promotes sodium retention and is frequently elevated in patients with resistant hypertension (59, 78). Future studies should investigate whether genetic variants affecting CK2 or Ank-3 expression or function are associated with salt-sensitive hypertension in human populations.

While our study provides compelling evidence for the physiological importance of CK2/Ank-3/ENaC signaling, there are some limitations. First, blood pressure was not directly measured in our KO models. While the robust natriuretic phenotype strongly suggests that CK2/Ank-3-dependent ENaC regulation would impact blood pressure under conditions of chronic sodium excess, compensatory mechanisms (RAAS activation, increased proximal reabsorption, and sympathetic adjustments) may maintain blood pressure within the normal range under standard laboratory conditions, as observed in other ENaC-regulatory knockout models such as SGK1 KO mice (79). Future studies incorporating telemetric blood pressure monitoring under normal and high-salt diets are warranted. Second, we have not examined whether CK2/Ank-3 coupling is altered in hypertensive disease models. Studies in Dahl salt-sensitive rats, spontaneously hypertensive rats, or aldosterone-infusion models could reveal whether this pathway is dysregulated in hypertension and whether its manipulation affects blood pressure. Additionally, while we focused on the β-ENaC subunit because it contains the most prominent CK2 consensus sites, the γ subunit could also be CK2 substrate (38). Comprehensive analysis of CK2 phosphorylation across all three ENaC subunits could reveal additional regulatory complexity.

In conclusion, our study establishes CK2-dependent phosphorylation and Ank-3-dependent scaffolding as essential determinants of ENaC trafficking, membrane retention, and activity in renal cells. Disruption of this pathway reduces ENaC activity and drives accelerated Na^+^ excretion, demonstrating that CK2/Ank-3 coupling is physiologically required for normal Na^+^ homeostasis. Future work clarifying how this pathway integrates with hormonal regulation, cytoskeletal dynamics, and other post-translational modifications will provide a more complete picture of ENaC regulation and may identify additional therapeutic strategies for improving blood pressure control.

## Materials and methods

### Animal care and use

All procedures involving animals were conducted in accordance with the National Institutes of Health guidelines for the care and use of laboratory animals. All animal protocols were reviewed and approved by the Institutional Animal Care and Use Committee at the University of Texas Health Science Center at San Antonio (UTHSCSA). Animals were housed and maintained in the Laboratory Animal Resources facility at UTHSCSA, a USDA-licensed and AAALAC-accredited vivarium, under standard temperature, humidity, and light/dark cycle conditions. All in vivo experiments were carried out in accordance with ARRIVE recommendations.

### Generation of collecting duct principal cell-specific CK2 and Ank-3 knockout mice

Principal cell-specific knockout mice for CSNK2A1 (CK2α) and Ank-3 were generated using Cre-loxP recombination under control of the Aqp2 promoter, which restricts recombination to collecting duct principal cells (60, 61). The principal cell-specific CK2 knockout mouse was generated by breeding male CSNK2A1 floxed mice with female B6.Cg-Tg(Aqp2-Cre)1Dek/J mice. The CK2α conditional allele (CSNK2A1^fl/fl^) was generated by insertion of loxP sites flanking exon 2 of the CSNK2A1 locus, which encodes the ATP-binding pocket and the dominant CK2α catalytic activity. Deletion of exon 2 produces a frameshift and premature stop codon, resulting in functional ablation of CK2α. The CSNK2A1^fl/fl^ line (provided by H. Rebholz, Rockefeller University, New York, NY, USA) has been previously characterized (49). Cre expression under the Aqp2 promoter selectively excises the catalytic α subunit of CK2 in collecting duct principal cells while leaving the α′ and β isoforms intact in other cell types. The B6.Cg-Tg(Aqp2-cre)1Dek/J mouse was a kind gift from Dr. D. Kohan (University of Utah Health Sciences Center, Salt Lake City, UT, USA) and has been described previously (60, 61). Offspring homozygous for the floxed CSNK2A1 allele and for Aqp2-Cre (CSNK2A1^fl/fl^;Aqp2-Cre) were designated as principal cell-specific CK2 knockout (PC-CK2 KO) animals and were used for experiments. Littermates lacking the Cre transgene, as well as Aqp2-Cre negative littermates lacking floxed CSNK2A1 alleles, were used as controls. Genotyping of the CSNK2A1 floxed allele was performed using PCR with the following primers: forward 5′-GAGTCATCTGTCATCTGTAGC-3′ and reverse 5′-GACCACATCCTAACTATCC-3′, yielding a 550 bp product. Aqp2-Cre was detected using the standard primer set (forward 5′-CTCTGCAGGAACTGGTGCTGG-3′; reverse 5′-GCGAACATCTTCAGGTTCTGCGG-3′), producing a 673 bp amplicon.

The principal cell-specific Ank-3 KO mouse was generated as previously described (44), by crossing floxed Ank-3 mice (kind gift from Dr. V. Bennett, HHMI/Duke University, NC) with the Aqp2-Cre driver line. Cre-positive, homozygous floxed Ank-3 mice were used as experimental animals. Cre-negative littermates with or without floxed Ank-3 alleles were used as controls.

All strains were maintained on a C57BL/6 background by continued breeding of floxed:Cre-positive females to floxed males. Mice used in experiments were between 8 and 16 weeks old. Routine PCR-based genotyping was performed on genomic DNA obtained from tail biopsies using allele-specific primers for the floxed and Aqp2-Cre alleles. KO animals displayed no abnormalities in viability, gross appearance, or body weight compared with control littermates. Both male and female mice were included in similar proportions across all experiments. No significant differences were observed between male and female mice of either genotype. All experiments were performed using mice maintained on a standard control diet unless otherwise specified.

### Cell culture, ENaC expression constructs, and site-directed mutagenesis

COS-7 cells (African green monkey kidney fibroblast-like line) and CHO cells (Chinese hamster ovary) were obtained from ATCC (Manassas, VA, USA). COS-7 were maintained in Dulbecco's modified Eagle's medium supplemented with 10% fetal bovine serum and 1% penicillin–streptomycin. CHO were maintained in Ham's F-12 K (Kaighn's) medium supplemented with 10% FBS and 1% P/S. All ells were kept at 37 °C in a 5% CO_2_ humidified incubator.

Site-directed mutagenesis of the pEYFP-C1-βENaC plasmid was performed using the QuickChange Lightning Site-Directed Mutagenesis Kit (Agilent Technologies, Santa Clara, CA, USA). Mutagenic primers were designed using the Agilent online primer design platform to introduce alanine substitutions within discrete clusters of CK2 consensus residues and Ank-3 binding residues in the β-ENaC C-terminal domain. Three mutant constructs were generated: βS631A/S633A/E634A/E636A-ENaC (CK2 binding sites), βP625A/L626A/E630A-ENaC (Ank-3 binding sites), and βP625A/L626A/E630A/S631A/S633A/E634A/E636A-ENaC (combined CK2/Ank-3 binding sites). Standard desalinated forward and reverse primers were used for PCR amplification. The CK2 multisite mutant (S631A/S633A/E634A/E636A) was generated using a single forward primer (CK2-Fwd: 5′-GACACCATGGAGGCGGACGCTGCGGTGGCGGCCATCTAGGA-3′). To create the Ank-3 mutant (P625A/L626A/E630A), a two-step method was used: first, the E630A mutant was introduced into the WT-βENaC plasmid using primer E630A-Fwd (5′-GCTGGACACCATGGCGTCGGACAGTGAGG-3′), followed by the addition of P625A and L626A mutations using primers AnkG630-Fwd (5′-CCTGAGGCTGCAGGCGGCGGACACCATGGCG-3′) and AnkG630-Rev (5′-GCGCATGGTGTCCGCCGCCTGCAGCCTCAGG-3′). The combined CK2/Ank-3 multisite mutant (P625A/L626A/E630A/S631A/S633A/E634A/E636A) was generated in two steps: first, E630A was introduced into the CK2 multisite mutant plasmid using primer E630A2-Fwd (5′-TGGACACCATGGCGGCGGACGCTGC-3′), and then the AnkG630-Fwd and AnkG630-Rev primers were used to add the P625A and L626A mutations. After PCR amplification, all reactions were treated with DpnI to digest parental methylated plasmid DNA, and the products were purified using a PCR clean-up kit (Zymo Research, Irvine, CA, USA) prior to transformation into TOP10 competent cells (Invitrogen). All constructs were confirmed by Sanger sequencing (Psomagen, Rockville, MD, USA) before use. For CK2 gain-of-function, expression plasmids encoding CK2α (pZW6, Addgene #27086, Watertown, MA, USA) and CK2β (pZW12, Addgene #27088) were used. For CK2 loss-of-function, cells were treated with TBB (4,5,6,7-tetrabromobenzotriazole) at a final concentration of 200 nM for 30 min prior to the start of the experiment.

### TIRF-FRAP imaging

Total internal reflection fluorescence microscopy (TIRF) was used to selectively excite fluorophores at the plasma membrane in COS-7 cells expressing eYFP-tagged ENaC subunits, as previously described (44, 80). The optical configuration limited excitation to the thin region adjacent to the glass-cell interface, producing an evanescent field that decays exponentially with distance and thereby illuminates only approximately the first ∼100 nm of the cell contacting the coverslip. This approach allows detection of surface-localized ENaC with minimal contribution from intracellular pools. Cells were imaged at room temperature using a Nikon TE2000 inverted microscope (Nikon Instruments, Melville, NY, USA) configured for through-the-lens prism-less TIRF. The system was mounted on an active vibration-isolation table (Technical Manufacturing Corp., Peabody, MA, USA) to minimize mechanical drift. Excitation of eYFP was achieved using the 514-nm line of an argon ion laser. An acoustic-optic tunable filter (Prairie Technologies, Hutto, TX, USA) was used to restrict excitation to 514 nm, and a 514 nm dichroic mirror (Z514rdc; Chroma Technology, Bellows Falls, VT, USA) in combination with Z514/10× excitation and HQ560/50 m emission filters were used. Fluorescence signals were collected through a Plan Apo TIRF 60× 1.45 NA oil-immersion objective (Nikon) and detected with a cooled 16-bit EMCCD camera (Cascade 512F; Roper Scientific, Sarasota, FL, USA). Image acquisition and processing were controlled using MetaMorph software (Molecular Devices, San Jose, CA, USA). Exposure time was 200 ms for all acquisition timepoints.

Photobleaching was performed directly under TIRF illumination using the 514-nm argon laser at full output (100%) for 10 s. Images were collected prior to photobleaching, immediately following bleach, and then at 1-min intervals throughout the recovery period. Laser power, detector gain, and exposure settings were held constant for the entirety of each experiment except during the 10-s bleaching pulse. TBB treatment, CK2 overexpression, or β-ENaC mutation status were the only variables altered between experimental conditions.

### FRET microscopy

Fluorescence resonance energy transfer (FRET) experiments were performed in COS-7 cells transfected with eYFP-tagged β-ENaC constructs (WT or β_Ank-3-site) together with eCFP-Ank-3 using PolyFect (Qiagen, Valencia, CA, USA) according to the manufacturer's instructions and previously described (81). Imaging was performed on a Zeiss LSM 510 laser-scanning confocal microscope (Zeiss, Thornwood, NY, USA) using a 60×/1.4 NA oil-immersion objective. Donor (eCFP) and acceptor (eYFP) were excited at 458 and 514 nm, respectively. Emission was collected with 468.5–505.5 nm band-pass filters for eCFP (HQ 487/37; Chroma, Brattleboro, VT, USA) and 525–575 nm band-pass filters for eYFP (HQ 550/50; Chroma). An NFT 515 nm dichroic beamsplitter directed donor and acceptor signals to separate PMTs. Acquisition settings (laser power, gain, pinhole) were kept constant across conditions.

Acceptor photobleaching (donor dequenching) was used to quantify FRET. A defined ROI was photobleached at 514 nm to reduce eYFP fluorescence to <15% of prebleach intensity. Images were collected immediately before and after bleaching under identical settings. Percent FRET efficiency was calculated as:


$$\text{\%}\mathrm{FRET}=\left[\frac{\left(A_{1}-A_{0})\times (B_{0}/B_{1}\right)}{A_{0}}\right]\times 100\text{\%},$$

where *A*_0_ and *A*_1_ are donor (eCFP) intensities in the bleached ROI before and after photobleaching, and *B*_0_ and *B*_1_ are donor intensities in a nonbleached reference ROI before and after photobleaching, respectively. This normalization controls nonspecific changes in donor signals. All analyses were performed on fixed cells prepared and imaged under identical conditions.

### Whole-cell patch-clamp electrophysiology

CHO cells were seeded onto 0.01% poly-L-lysine coated glass slips (Sigma, St. Louis, MO, USA) and grown to ∼60% confluence. Cells were then transfected with plasmids encoding mouse α-, β-, and γ-ENaC subunits genetically linked N-terminal eCFP tags (81, 82) using PolyFect transfection reagent (Qiagen, Valencia, CA, USA) according to the manufacturer's instructions. For all ENaC expression conditions, a total of 2 µg plasmid cDNA was used per 35-mm dish. In addition to WT ENaC, cells were transfected in parallel with β-ENaC constructs containing targeted alanine substitutions in the CK2 site (β_CK2-site ENaC), Ank-3 site (β_Ank-3-site ENaC), or both sites (β_CK2+Ank-3-site ENaC). Cells were maintained in the transfection mixture for 4–5 h, after which the medium was replaced with fresh culture medium. Electrophysiological recordings were performed 24–48 h after transfection. Whole-cell ENaC currents were recorded at room temperature using standard methods (83). The external solution contained (in mM): 150 NaCl, 1 CaCl_2_, 2 MgCl_2_, and 10 HEPES, adjusted to pH 7.4. The intracellular solution contained (in mM): 120 CsCl, 5 NaCl, 2 MgCl_2_, 5 EGTA, 10 HEPES, 2 ATP-Mg, and 0.1 GTP-Na_2_, adjusted to pH 7.4. Recordings were performed using an Axopatch-200B amplifier connected with a Digidata-1550B digitizer and controlled by pClamp 11 (Molecular Devices). All currents were filtered at 1kHz. Cells were voltage clamped at a holding potential of +40 mV, and whole-cell current was elicited using 500 ms voltage ramps from +60 mV to −100 mV. ENaC-dependent current was defined as the inward Na^+^ current blocked by 10 µM amiloride added to the extracellular solution.

### Isolation of CNT/CCD tubules and cell-attached patch clamp recording

Single-channel recordings from native ENaC in principal cells were made using freshly isolated segments of connecting tubule/cortical collecting duct (CNT/CCD) as previously described (84). Briefly, kidneys from PC-CK2 KO mice, PC-Ank-3 KO mice, and corresponding littermate controls were removed and cut into transverse slices. Individual CNT/CCD segments were micro dissected under a stereomicroscope using fine forceps and mounted onto 0.01% poly-L-lysine coated glass slips. Mounted tubules were placed on the stage of an inverted microscope and mechanically opened by gently teasing apart the tubular wall with sharpened glass pipettes to expose the luminal membrane surface of individual principal cells. Patch clamp recordings were performed in the cell-attached mode under continuous superfusing with external solution containing (in mM): 150 NaCl, 5 KCl, 1 CaCl_2_, 2 MgCl_2_, 5 glucose, and 10 HEPES, adjusted to pH 7.4. Patch pipettes were filled with solution containing (in mM): 140 LiCl, 2 MgCl_2_, and 10 HEPES, adjusted to pH 7.4. In experiments testing CK2 inhibition, tubules were preincubated in 200 nM TBB for 30 min prior to seal formation and maintained in the inhibitor throughout recording. Channel activity was recorded in gap-free mode, digitized, and analyzed as the product of the number of active channels (N) and their open probability (P_0_) to yield NP_0_.

### Metabolic cage studies

Metabolic balance experiments were conducted in individual metabolic cages (Tecniplast, Buguggiate, Italy) using PC-CK2 KO mice, PC-Ank-3 KO mice, and their littermate controls. Mice were ∼3-month-old (25 g body weight) and both sexes were used in similar proportions. Protocols were adapted from our previously established protocols with minor modifications (44, 85). Mice had unrestricted access to standard chow and water during an initial 3-day acclimation period. Following acclimation, mice received a single intraperitoneal injection of 100 µL sterile 0.9% NaCl (acute Na^+^ load). After this injection, urine was collected at 3-h intervals over 9 h and urinary Na^+^ concentration and volume were quantified. From these measurements, U_Na_V was calculated, and cumulative Na^+^ excretion was expressed as the percentage of the administered Na^+^ load. In a separate set of experiments, after the 3-day acclimation period on standard chow, mice were transitioned to a sodium-deficient diet (0.01% Na^+^, TEKLAD custom diet TD 90228; Envigo, Indianapolis, IN, USA) and maintained on this diet for 3 days. Mice were then housed in metabolic cages, and urine was collected at 0, 24, 48, and 72 h. At the 24 h collection time point, animals received a single intraperitoneal injection of benzamil (1.4 mg/kg) dissolved in 100 µL sterile saline. At each interval, urine volume and urinary Na^+^ concentration were recorded. Urinary Na^+^ concentration was determined using a flame photometer (Jenway, Staffordshire, UK). For baseline assessment, mice maintained on standard chow were housed in metabolic cages and urine was collected over 3 h for measurement of urine volume and urinary Na^+^ and K^+^. Mice were then anesthetized and euthanized, and blood was collected for plasma separation. Plasma and urinary Na^+^ and K^+^ were measured by flame photometer, and plasma Cl^−^ using the QuantiChrom Chloride Assay Kit (DICL-250; BioAssay Systems, Hayward, CA, USA). Serum creatinine and BUN were determined using the Creatinine Assay Kit (ab65340; Abcam, Cambridge, UK) and the Blood Urea Nitrogen Colorimetric Detection Kit (EIABUN; Invitrogen/Thermo Fisher Scientific, Waltham, MA, USA), respectively. Plasma aldosterone and cystatin C were measured using the Aldosterone ELISA Kit (501090; Cayman Chemical, Ann Arbor, MI, USA) and the Mouse Cystatin C ELISA Kit (80738; Crystal Chem, Elk Grove Village, IL, USA).

### Statistical analysis

All experiments were performed with at least three independent biological replicates unless otherwise specified. Numerical data are presented as mean ± SEM. Statistical comparisons between two groups were performed using unpaired Student's t test, while comparisons among multiple groups used one-way ANOVA followed by appropriate post hoc tests. Statistical analyses were performed using GraphPad Prism 10 software (GraphPad Software, San Diego, CA, USA). Statistical significance was assigned at *P* ≤ 0.05.

## Supplementary Material

pgag253_Supplementary_Data

## Data Availability

All data supporting the findings of this study are available within the article and its Supplementary Information files.

## References

[1] Mills KT, Stefanescu A, He J. 2020. The global epidemiology of hypertension. Nat Rev Nephrol. 16:223–237. 32024986 10.1038/s41581-019-0244-2 PMC7998524

[2] Burnier M, Damianaki A. 2023. Hypertension as cardiovascular risk factor in chronic kidney disease. Circ Res. 132:1050–1063. 37053276 10.1161/CIRCRESAHA.122.321762

[3] Cushman WC . 2003. The burden of uncontrolled hypertension: morbidity and mortality associated with disease progression. J Clin Hypertens (Greenwich). 5:14–22. 12826766 10.1111/j.1524-6175.2003.02464.x PMC8099352

[4] Pacholko A, Iadecola C. 2024. Hypertension, neurodegeneration, and cognitive decline. Hypertension. 81:991–1007. 38426329 10.1161/HYPERTENSIONAHA.123.21356 PMC11023809

[5] Egan BM, Kjeldsen SE, Grassi G, Esler M, Mancia G. 2019. The global burden of hypertension exceeds 1.4 billion people: should a systolic blood pressure target below 130 become the universal standard? J Hypertens. 37:1148–1153. 30624370 10.1097/HJH.0000000000002021

[6] NCD Risk Factor Collaboration (NCD-RisC) . 2021. Worldwide trends in hypertension prevalence and progress in treatment and control from 1990 to 2019: a pooled analysis of 1201 population-representative studies with 104 million participants. Lancet. 398:957–980. 34450083 10.1016/S0140-6736(21)01330-1 PMC8446938

[7] Mills KT, et al 2016. Global disparities of hypertension prevalence and control: a systematic analysis of population-based studies from 90 countries. Circulation. 134:441–450. 27502908 10.1161/CIRCULATIONAHA.115.018912 PMC4979614

[8] Oparil S, et al 2018. Hypertension. Nat Rev Dis Primers. 4:18014. 29565029 10.1038/nrdp.2018.14 PMC6477925

[9] Te Riet L, van Esch JHM, Roks AJM, van den Meiracker AH, Danser AHJ. 2015. Hypertension. Circ Res. 116:960–975. 25767283 10.1161/CIRCRESAHA.116.303587

[10] Grillo A, Salvi L, Coruzzi P, Salvi P, Parati G. 2019. Sodium intake and hypertension. Nutrients. 11:1970. 31438636 10.3390/nu11091970 PMC6770596

[11] He FJ, Tan M, Ma Y, MacGregor GA. 2020. Salt reduction to prevent hypertension and cardiovascular disease: JACC state-of-the-art review. J Am Coll Cardiol. 75:632–647. 32057379 10.1016/j.jacc.2019.11.055

[12] Egan BM, et al 2025. PERSPECTIVE—the growing global benefits of limiting salt intake: an urgent call from the World Hypertension League for more effective policy and public health initiatives. J Hum Hypertens. 39:241–245. 40119141 10.1038/s41371-025-00990-1 PMC11985337

[13] Elijovich F, et al 2016. Salt sensitivity of blood pressure: a scientific statement from the American Heart Association. Hypertension. 68:e7–e46. 27443572 10.1161/HYP.0000000000000047

[14] Hummler E . 1999. Implication of ENaC in salt-sensitive hypertension. J Steroid Biochem Mol Biol. 69:385–390. 10419016 10.1016/s0960-0760(99)00073-4

[15] Rossier BC . 2014. Epithelial sodium channel (ENaC) and the control of blood pressure. Curr Opin Pharmacol. 15:33–46. 24721652 10.1016/j.coph.2013.11.010

[16] Mutchler SM, Kirabo A, Kleyman TR. 2021. Epithelial sodium channel and salt-sensitive hypertension. Hypertension. 77:759–767. 33486988 10.1161/HYPERTENSIONAHA.120.14481 PMC7878349

[17] Canessa CM, et al 1994. Amiloride-sensitive epithelial Na+ channel is made of three homologous subunits. Nature. 367:463–467. 8107805 10.1038/367463a0

[18] Noreng S, Bharadwaj A, Posert R, Yoshioka C, Baconguis I. 2018. Structure of the human epithelial sodium channel by cryo-electron microscopy. Elife. 7:e39340. 30251954 10.7554/eLife.39340 PMC6197857

[19] Firsov D, Gautschi I, Merillat AM, Rossier BC, Schild L. 1998. The heterotetrameric architecture of the epithelial sodium channel (ENaC). EMBO J. 17:344–352. 9430626 10.1093/emboj/17.2.344 PMC1170385

[20] Shimkets RA, et al 1994. Liddle's syndrome: heritable human hypertension caused by mutations in the beta subunit of the epithelial sodium channel. Cell. 79:407–414. 7954808 10.1016/0092-8674(94)90250-x

[21] Hansson JH, et al 1995. Hypertension caused by a truncated epithelial sodium channel gamma subunit: genetic heterogeneity of Liddle syndrome. Nat Genet. 11:76–82. 7550319 10.1038/ng0995-76

[22] Chang SS, et al 1996. Mutations in subunits of the epithelial sodium channel cause salt wasting with hyperkalaemic acidosis, pseudohypoaldosteronism type 1. Nat Genet. 12:248–253. 8589714 10.1038/ng0396-248

[23] Hanukoglu A . 1991. Type I pseudohypoaldosteronism includes two clinically and genetically distinct entities with either renal or multiple target organ defects. J Clin Endocrinol Metab. 73:936–944. 1939532 10.1210/jcem-73-5-936

[24] Butterworth MB . 2010. Regulation of the epithelial sodium channel (ENaC) by membrane trafficking. Biochim Biophys Acta. 1802:1166–1177. 20347969 10.1016/j.bbadis.2010.03.010 PMC2921481

[25] Staub O, et al 1996. WW domains of Nedd4 bind to the proline-rich PY motifs in the epithelial Na+ channel deleted in Liddle's syndrome. EMBO J. 15:2371–2380. 8665844 PMC450167

[26] Zhou R, Patel SV, Snyder PM. 2007. Nedd4-2 catalyzes ubiquitination and degradation of cell surface ENaC. J Biol Chem. 282:20207–20212. 17502380 10.1074/jbc.M611329200

[27] Debonneville C, et al 2001. Phosphorylation of Nedd4-2 by Sgk1 regulates epithelial Na(+) channel cell surface expression. EMBO J. 20:7052–7059. 11742982 10.1093/emboj/20.24.7052 PMC125341

[28] Snyder PM, Steines JC, Olson DR. 2004. Relative contribution of Nedd4 and Nedd4-2 to ENaC regulation in epithelia determined by RNA interference. J Biol Chem. 279:5042–5046. 14645220 10.1074/jbc.M312477200

[29] Ichimura T, et al 2005. 14-3-3 proteins modulate the expression of epithelial Na+ channels by phosphorylation-dependent interaction with Nedd4-2 ubiquitin ligase. J Biol Chem. 280:13187–13194. 15677482 10.1074/jbc.M412884200

[30] Litchfield DW . 2003. Protein kinase CK2: structure, regulation and role in cellular decisions of life and death. Biochem J. 369:1–15. 12396231 10.1042/BJ20021469 PMC1223072

[31] Niefind K, Guerra B, Ermakowa I, Issinger OG. 2001. Crystal structure of human protein kinase CK2: insights into basic properties of the CK2 holoenzyme. EMBO J. 20:5320–5331. 11574463 10.1093/emboj/20.19.5320 PMC125641

[32] Vilk G, Saulnier RB, Pierre RS, Litchfield DW. 1999. Inducible expression of protein kinase CK2 in mammalian cells. Evidence for functional specialization of CK2 isoforms. J Biol Chem. 274:14406–14414. 10318865 10.1074/jbc.274.20.14406

[33] Brachet A, et al 2010. Ankyrin G restricts ion channel diffusion at the axonal initial segment before the establishment of the diffusion barrier. J Cell Biol. 191:383–395. 20956383 10.1083/jcb.201003042 PMC2958486

[34] Pan Z, et al 2006. A common ankyrin-G-based mechanism retains KCNQ and NaV channels at electrically active domains of the axon. J Neurosci. 26:2599–2613. 16525039 10.1523/JNEUROSCI.4314-05.2006 PMC6675151

[35] Lemaillet G, Walker B, Lambert S. 2003. Identification of a conserved ankyrin-binding motif in the family of sodium channel alpha subunits. J Biol Chem. 278:27333–27339. 12716895 10.1074/jbc.M303327200

[36] Bennett V, Lorenzo DN. 2016. An adaptable spectrin/ankyrin-based mechanism for long-range organization of plasma membranes in vertebrate tissues. Curr Top Membr. 77:143–184. 26781832 10.1016/bs.ctm.2015.10.001

[37] Klemens CA, Edinger RS, Kightlinger L, Liu X, Butterworth MB. 2017. Ankyrin G expression regulates apical delivery of the epithelial sodium channel (ENaC). J Biol Chem. 292:375–385. 27895120 10.1074/jbc.M116.753616 PMC5217695

[38] Bachhuber T, et al 2008. Regulation of the epithelial Na+ channel by the protein kinase CK2. J Biol Chem. 283:13225–13232. 18308722 10.1074/jbc.M704532200 PMC3259572

[39] Berman JM, Mironova E, Stockand JD. 2018. Physiological regulation of the epithelial Na(+) channel by casein kinase II. Am J Physiol Renal Physiol. 314:F367–F372. 29021227 10.1152/ajprenal.00469.2017 PMC5899227

[40] Soundararajan R, Lu M, Pearce D. 2012. Organization of the ENaC-regulatory machinery. Crit Rev Biochem Mol Biol. 47:349–359. 22506713 10.3109/10409238.2012.678285 PMC4319811

[41] Soundararajan R, et al 2012. Scaffold protein connector enhancer of kinase suppressor of Ras isoform 3 (CNK3) coordinates assembly of a multiprotein epithelial sodium channel (ENaC)-regulatory complex. J Biol Chem. 287:33014–33025. 22851176 10.1074/jbc.M112.389148 PMC3463358

[42] Kleyman TR, Eaton DC. 2020. Regulating ENaC's gate. Am J Physiol Cell Physiol. 318:C150–C162. 31721612 10.1152/ajpcell.00418.2019 PMC6985836

[43] Shi H, et al 2002. Casein kinase 2 specifically binds to and phosphorylates the carboxy termini of ENaC subunits. Eur J Biochem. 269:4551–4558. 12230567 10.1046/j.1432-1033.2002.03154.x

[44] Abd El-Aziz TM, et al 2021. Mechanisms and consequences of casein kinase II and ankyrin-3 regulation of the epithelial Na+ channel. Sci Rep. 11:14600. 34272444 10.1038/s41598-021-94118-3 PMC8285517

[45] Xu M, Cooper EC. 2015. An ankyrin-G N-terminal gate and protein kinase CK2 dually regulate binding of voltage-gated sodium and KCNQ2/3 potassium channels. J Biol Chem. 290:16619–16632. 25998125 10.1074/jbc.M115.638932 PMC4505415

[46] Vacher H, Trimmer JS. 2012. Trafficking mechanisms underlying neuronal voltage-gated ion channel localization at the axon initial segment. Epilepsia. 53 Suppl 9:21–31. 23216576 10.1111/epi.12032 PMC3531813

[47] Ceglia I, Flajolet M, Rebholz H. 2011. Predominance of CK2α over CK2α’ in the mammalian brain. Mol Cell Biochem. 356:169–175. 21761202 10.1007/s11010-011-0963-6

[48] Rebholz H, et al 2009. CK2 negatively regulates Galphas signaling. Proc Natl Acad Sci U S A. 106:14096–14101. 19666609 10.1073/pnas.0906857106 PMC2729026

[49] Rebholz H, Zhou M, Nairn AC, Greengard P, Flajolet M. 2013. Selective knockout of the casein kinase 2 in d1 medium spiny neurons controls dopaminergic function. Biol Psychiatry. 74:113–121. 23290496 10.1016/j.biopsych.2012.11.013 PMC3878430

[50] Rossi M, et al 2015. CK2 acts as a potent negative regulator of receptor-mediated insulin release in vitro and in vivo. Proc Natl Acad Sci U S A. 112:E6818–E6824. 26598688 10.1073/pnas.1519430112 PMC4679045

[51] Peters LL, et al 1995. Ank3 (epithelial ankyrin), a widely distributed new member of the ankyrin gene family and the major ankyrin in kidney, is expressed in alternatively spliced forms, including forms that lack the repeat domain. J Cell Biol. 130:313–330. 7615634 10.1083/jcb.130.2.313 PMC2199924

[52] Bennett V, Healy J. 2009. Membrane domains based on ankyrin and spectrin associated with cell-cell interactions. Cold Spring Harb Perspect Biol. 1:a003012. 20457566 10.1101/cshperspect.a003012 PMC2882121

[53] Nilsson KR Jr, Bennett V. 2009. Ankyrin-based patterning of membrane microdomains: new insights into a novel class of cardiovascular diseases. J Cardiovasc Pharmacol. 54:106–115. 19636256 10.1097/FJC.0b013e3181b2b6ed

[54] Bennett V, Lorenzo DN. 2013. Spectrin- and ankyrin-based membrane domains and the evolution of vertebrates. Curr Top Membr. 72:1–37. 24210426 10.1016/B978-0-12-417027-8.00001-5

[55] He M, Abdi KM, Bennett V. 2014. Ankyrin-G palmitoylation and βII-spectrin binding to phosphoinositide lipids drive lateral membrane assembly. J Cell Biol. 206:273–288. 25049274 10.1083/jcb.201401016 PMC4107783

[56] Jenkins PM, He M, Bennett V. 2015. Dynamic spectrin/ankyrin-G microdomains promote lateral membrane assembly by opposing endocytosis. Sci Adv. 1:e1500301. 26523289 10.1126/sciadv.1500301 PMC4624203

[57] Qu F, et al 2016. Ankyrin-B is a PI3P effector that promotes polarized α5β1-integrin recycling via recruiting RabGAP1L to early endosomes. Elife. 5:e20417. 27718357 10.7554/eLife.20417 PMC5089861

[58] Zhou D, et al 1998. Ankyring is required for clustering of voltage-gated Na channels at axon initial segments and for normal action potential firing. J Cell Biol. 143:1295–1304. 9832557 10.1083/jcb.143.5.1295 PMC2133082

[59] Edinger RS, et al 2014. Aldosterone regulates microRNAs in the cortical collecting duct to alter sodium transport. J Am Soc Nephrol. 25:2445–2457. 24744440 10.1681/ASN.2013090931 PMC4214524

[60] Nelson RD, et al 1998. Expression of an AQP2 Cre recombinase transgene in kidney and male reproductive system of transgenic mice. Am J Physiol. 275:C216–C226. 9688853 10.1152/ajpcell.1998.275.1.C216

[61] Stricklett PK, Nelson RD, Kohan DE. 1999. Targeting collecting tubules using the aquaporin-2 promoter. Exp Nephrol. 7:67–74. 9892817 10.1159/000020587

[62] Montenarh M, Götz C. 2020. Protein kinase CK2 and ion channels (Review). Biomed Rep. 13:55. 10.3892/br.2020.1362 PMC7560519 33082952

[63] Bréchet A, et al 2008. Protein kinase CK2 contributes to the organization of sodium channels in axonal membranes by regulating their interactions with ankyrin G. J Cell Biol. 183:1101–1114. 19064667 10.1083/jcb.200805169 PMC2600743

[64] Escobedo G, Wu Y, Ogawa Y, Ding X, Rasband MN. 2024. An evolutionarily conserved AnkyrinG-dependent motif clusters axonal K2P K+ channels. J Cell Biol. 223:e202401140. 39078369 10.1083/jcb.202401140 PMC11289519

[65] Haitin Y, Attali B. 2008. The C-terminus of Kv7 channels: a multifunctional module. J Physiol. 586:1803–1810. 18218681 10.1113/jphysiol.2007.149187 PMC2375714

[66] Meggio F, Pinna LA. 2003. One-thousand-and-one substrates of protein kinase CK2? FASEB J. 17:349–368. 12631575 10.1096/fj.02-0473rev

[67] Wang C, et al 2014. Structural basis of diverse membrane target recognitions by ankyrins. eLife. 3:e04353. 25383926 10.7554/eLife.04353 PMC4358367

[68] Cunha SR, Mohler PJ. 2009. Ankyrin protein networks in membrane formation and stabilization. J Cell Mol Med. 13:4364–4376. 19840192 10.1111/j.1582-4934.2009.00943.x PMC4515052

[69] Ronzaud C, et al 2007. Impairment of sodium balance in mice deficient in renal principal cell mineralocorticoid receptor. J Am Soc Nephrol. 18:1679–1687. 17475815 10.1681/ASN.2006090975

[70] Palmer LG, Schnermann J. 2015. Integrated control of Na transport along the nephron. Clin J Am Soc Nephrol. 10:676–687. 25098598 10.2215/CJN.12391213 PMC4386267

[71] Guyton AC . 1991. Blood pressure control–special role of the kidneys and body fluids. Science. 252:1813–1816. 2063193 10.1126/science.2063193

[72] Welling PA, Ho K. 2009. A comprehensive guide to the ROMK potassium channel: form and function in health and disease. Am J Physiol Renal Physiol. 297:F849–F863. 19458126 10.1152/ajprenal.00181.2009 PMC2775575

[73] Woda CB, Bragin A, Kleyman TR, Satlin LM. 2001. Flow-dependent K+ secretion in the cortical collecting duct is mediated by a maxi-K channel. Am J Physiol Renal Physiol. 280:F786–F793. 11292620 10.1152/ajprenal.2001.280.5.F786

[74] Rieg T, et al 2007. The role of the BK channel in potassium homeostasis and flow-induced renal potassium excretion. Kidney Int. 72:566–573. 17579662 10.1038/sj.ki.5002369

[75] Liu W, Morimoto T, Woda C, Kleyman TR, Satlin LM. 2007. Ca2+ dependence of flow-stimulated K secretion in the mammalian cortical collecting duct. Am J Physiol Renal Physiol. 293:F227–F235. 17389680 10.1152/ajprenal.00057.2007

[76] Pluznick JL, Sansom SC. 2006. BK channels in the kidney: role in K(+) secretion and localization of molecular components. Am J Physiol Renal Physiol. 291:F517–F529. 16774904 10.1152/ajprenal.00118.2006

[77] Riepe FG . 2009. Clinical and molecular features of type 1 pseudohypoaldosteronism. Horm Res. 72:1–9. 10.1159/000224334 19571553

[78] Brown NJ . 2013. Contribution of aldosterone to cardiovascular and renal inflammation and fibrosis. Nat Rev Nephrol. 9:459–469. 23774812 10.1038/nrneph.2013.110 PMC3922409

[79] Wulff P, et al 2002. Impaired renal Na(+) retention in the sgk1-knockout mouse. J Clin Invest. 110:1263–1268. 12417564 10.1172/JCI15696 PMC151609

[80] Pochynyuk O, Staruschenko A, Bugaj V, LaGrange L, Stockand JD. 2007. Quantifying RhoA facilitated trafficking of the epithelial Na+ channel toward the plasma membrane with total internal reflection fluorescence-fluorescence recovery after photobleaching. J Biol Chem. 282:14576–14585. 17376773 10.1074/jbc.M701348200

[81] Staruschenko A, et al 2004. Fluorescence resonance energy transfer analysis of subunit stoichiometry of the epithelial Na^+^ channel. J Biol Chem. 279:27729–27734. 15096495 10.1074/jbc.M404169200

[82] Staruschenko A, Adams E, Booth RE, Stockand JD. 2005. Epithelial Na^+^ channel subunit stoichiometry. Biophys J. 88:3966–3975. 15821171 10.1529/biophysj.104.056804 PMC1305628

[83] Gamper N, Stockand JD, Shapiro MS. 2005. The use of Chinese hamster ovary (CHO) cells in the study of ion channels. J Pharmacol Toxicol Methods. 51:177–185. 15862463 10.1016/j.vascn.2004.08.008

[84] Mironova E, Bugay V, Pochynyuk O, Staruschenko A, Stockand JD. Recording Ion channels in isolated, split-opened tubules. In: Gamper N, editor. Ion channels: methods and protocols. Humana Press, 2013. p. 341–353. 10.1007/978-1-62703-351-0_27 PMC4181527 23529443

[85] Mironova E, et al 2015. Activation of ENaC by AVP contributes to the urinary concentrating mechanism and dilution of plasma. Am J Physiol Renal Physiol. 308:F237–F243. 25391898 10.1152/ajprenal.00246.2014 PMC4596725

