# Supplementary material for: ENaC phosphorylation facilitates ankyrin-3 interaction critical for renal sodium balance
Source: PNAS Nexus. 2026 Jul 29;5(8):pgag253. doi: 10.1093/pnasnexus/pgag253 (PMC13447671; doi:10.1093/pnasnexus/pgag253)
Supplement: Supplementary file 1 [file pgag253_supplementary_data.zip › pnasnexus_PNASNEXUS-2026-00439-TRR_Supporting_Information (1).docx]

**Supplementary Materials**

**Supplementary Table 1.**

| **Parameter** | **Units** | **WT**  **(n = 4)** | **PC-CK2 KO (n = 4)** | **PC-Ank-3 KO (n = 4)** |
| --- | --- | --- | --- | --- |
| **Plasma/Serum** | | | | |
| Sodium (Na⁺) | mmol/L | 161.4 ± 5.04 | 157.8 ± 3.89 | 152.6 ± 7.54 |
| Potassium (K⁺) | mmol/L | 3.7 ± 0.102 | 4.1 ± 0.097 | 3.9 ± 0.114 |
| Chloride (Cl⁻) | mmol/L | 114.8 ± 3.48 | 119.2 ± 5.03 | 121.7 ± 8.46 |
| Blood urea nitrogen (BUN) | mg/dL | 27.1 ± 0.95 | 30.4 ± 1.01 | 26.8 ± 0.98 |
| Creatinine | mg/dL | 0.241 ± 0.057 | 0.274 ± 0.041 | 0.259 ± 0.037 |
| Cystatin C | mg/L | 0.967 ± 0.081 | 1.03 ± 0.104 | 0.972 ± 0.098 |
| Aldosterone | pg/mL | 232 ± 18.16 | 409.1 ± 23.31*** | 414.3 ± 22.53*** |
| **Urine** | | | | |
| Urine volume | mL | 0.311 ± 0.021 | 0.383 ± 0.019 | 0.401 ± 0.025 |
| U_Na_V | nmol/min/g BW | 1.103 ± 0.047 | 1.142 ± 0.038 | 1.151 ± 0.048 |
| U_K_V | nmol/min/g BW | 3.209 ± 0.094 | 3.517 ± 0.102 | 3.432 ± 0.086 |

Data are displayed as mean ± SEM. Both male and female mice were used in similar proportions. Statistical significance for all data was assessed using 2-tailed unpaired Student’s *t*-tests (***P < 0.001).

**Figures legends**

**Supplementary Figure 1. CK2 inhibition with TBB abolishes mutation-dependent differences in β-ENaC trafficking and function.** **(A)** Representative TIRF-FRAP images of COS-7 cells pre-treated with TBB and expressing eYFP-ENaC variants, including WT β-ENaC (top row), the CK2-site mutant (second row), the Ank-3-site mutant (third row), and the CK2+Ank-3 double mutant (fourth row). Images show pre-bleach, 10 s after bleach, and 10 min after bleach. **(B)** Time-dependent FRAP curves at the plasma membrane for TBB-treated cells expressing WT eYFP-ENaC (black), the CK2-site mutant (red), the Ank-3-site mutant (blue), and the CK2+Ank-3 double mutant (green). Data represent mean recovery values from 5-6 cells across 2-3 independent transfections corresponding to the imaging conditions in panel (**A**). **(C)** Summary of FRAP values at 10 min for TBB-treated cells expressing WT and mutant eYFP-ENaC constructs. Individual data points are shown for WT (black), CK2-site mutants (red), Ank-3-site mutants (blue), and CK2+Ank-3 double mutants (green). Data are from the same experiments as panel (**A**). **(D)** Representative whole-cell current recordings from CHO cells treated with TBB and expressing WT mENaC, the CK2-site mutant, the Ank-3-site mutant, or the CK2+Ank-3 double mutant. Traces show currents before and after application of 10 µM amiloride (dotted lines). **(E)** Quantification of amiloride-sensitive ENaC current density (pA/pF) at -100 mV in TBB-treated CHO cells expressing WT (black), CK2-site mutant (red), Ank-3-site mutant (blue), or CK2+Ank-3 double mutant (green) channels. Each point represents an individual cell, with mean ± SEM indicated. Statistical significance for all data was assessed using 2-tailed unpaired Student’s *t*-tests (ns, P ≥ 0.05).

**Supplementary Figure 2. Proof-of-principle validation of the FRET assay used to assess βENaC-ank-3 interactions.** (**A**) Fluorescence micrographs of COS-7 cells co-expressing mCGY (a concatemer containing eCFP linked to eYFP by a 6-glycine spacer, positive control), mYFP with eCFP-Ank-3, eYFP-βENaC with mCFP, or eYFP-mt βENaC with mCFP, showing YFP and CFP channels before (first and second columns) and after (third and fourth columns) selective photobleaching of YFP. **(B)** Quantification of FRET efficiency for cells expressing the constructs shown in (**A**): mCGY (pink), mYFP+eCFP-Ank-3 (yellow), eYFP-βENaC+mCFP (purple), and eYFP-mt βENaC+mCFP (cyan). Data are from 8-19 cells per condition and are presented as mean ± SEM. Statistical significance for all data was assessed using 2-tailed unpaired Student’s *t*-tests (****P < 0.0001).

**Supplementary Figure 3. Consequences of principal-cell Ank-3 deletion for ENaC channel function in the collecting duct.** (**A**) Left, schematic of the breeding strategy used to generate PC-Ank-3 KO mice by crossing Ank-3^fl/fl^ animals with Aqp2-Cre transgenic mice. Right, representative gel containing PCR products from genotyping reactions for breeders (lanes 1 and 2) and PC-specific Ank-3 KO mice (lane 3). Bands corresponding to the Aqp2-Cre transgene and the floxed Ank-3 allele are indicated with arrowheads. The gel image was inverted (black to white) to enhance visual clarity without altering the underlying data. bp = base pairs. (**B, C**) Representative gap-free ENaC single-channel current recordings obtained in the cell-attached configuration from the apical membrane of principal cells in freshly isolated collecting duct tubules from WT littermates and PC-Ank-3 KO mice. Traces are shown under baseline conditions (top) and after pretreatment of isolated tubules with TBB (200 nM, 30 min; bottom). Inward Na^+^ current is downwards with dashed lines indicating the closed state. (**D-F**) Summary graph of ENaC activity (NPo), open probability (Po), and channel number (N) obtained from cell-attached recordings of WT littermate tubules and PC-Ank-3 KO tubules under baseline conditions (black and blue filled circles) and after TBB pretreatment (open black and open blue circles). Data represent 6-16 patches collected from 3-4 mice, corresponding to the recordings shown in panels (**B**) and (**C**). Both male and female mice were used in similar proportions. Statistical significance for all data was determined using 2-tailed unpaired Student’s *t*-test (ns, P ≥ 0.05; *P < 0.05; ***P < 0.001; ****P < 0.0001).

**Supplementary Figure 4. Principal-cell Ank-3 deletion enhances sodium load excretion *in vivo*.** (**A**) Cumulative Na⁺ excretion over a 9-hr period in WT mice (black filled circles, n = 7), WT mice treated with TBB (open black circles, n = 3), PC-Ank-3 KO mice (blue filled circles, n = 8), and PC-Ank-3 KO mice treated with TBB (open blue circles, n = 3). Mice received an intraperitoneal Na⁺ load (100 µL, 0.9% NaCl) at time 0, and cumulative Na⁺ excretion was measured every 3 hr. (**B**) Cumulative Na⁺ excretion at the 9-hr endpoint for the four experimental groups shown in panel (**A**). Data are plotted as mean ± SEM. (**C-F**) Summary graphs showing 24-hr urine volume (mL), urine osmolality (mmol/kg), urinary Na⁺ excretion (U_Na_V), and urinary K⁺ excretion (U_K_V) for WT littermates (black circles, n = 4) and PC-Ank-3 KO mice (blue circles, n = 4). Both male and female mice were used in similar proportions. Data are plotted as mean ± SEM. Statistical significance for all data was assessed using 2-tailed unpaired Student’s *t*-tests (ns, P ≥ 0.05; *P < 0.05; **P < 0.01).
